# Inflammatory Bowel Disease: From Multifactorial and Metabolic Pathogenesis to Systemic Disease and Precision Medicine

**DOI:** 10.3390/metabo16100710

**Published:** 2026-09-24

**Authors:** Vinícius Pedro Silva de Oliveira, Elizangela dos Anjos Silva, Silvio Pires Gomes

**Affiliations:** 1 Departamento de Cirurgia, Programa de Pós-Graduação em Anatomia Veterinária, Comparada e Translacional (PPG-AVCT), Faculdade de Medicina Veterinária e Zootecnia, Universidade de São Paulo, FMVZ/USP, Avenida Orlando Marques de Paiva, 87, São Paulo 05508-270, SP, Brazil; vinicius.ps.oliveira@usp.br; 2 Departamento de Cirurgia, Setor de Anatomia, Faculdade de Medicina Veterinária e Zootecnia, Universidade de São Paulo, FMVZ/USP, Avenida Orlando Marques de Paiva, 87, São Paulo 05508-270, SP, Brazil; 3 INBIO—Instituto de Biociências, UFMS—Universidade Federal do Mato Grosso do Sul, Campo Grande 79070-900, MS, Brazil; elizangela.anjos@ufms.br

**Keywords:** inflammatory bowel disease, Crohn’s disease, ulcerative colitis, gut microbiota, microbial metabolites, metabolic dysregulation, gut–liver axis, precision medicine

## Abstract

**Introduction:** Inflammatory bowel disease (IBD) is an umbrella term encompassing two major chronic immune-mediated disorders, Crohn’s disease (CD) and ulcerative colitis (UC). Its pathogenesis reflects complex interactions among genetic, microbial, metabolic, environmental, and immune factors. Growing evidence supports the concept of IBD as a systemic disease associated with intestinal dysbiosis, epithelial barrier dysfunction, metabolic alterations, and extraintestinal complications. However, these interconnected mechanisms and their systemic consequences are often examined separately, limiting an integrated understanding of IBD and its implications for individualized management. Accordingly, an updated synthesis linking intestinal, microbial, metabolic, immune, and systemic processes is needed to consolidate current evidence and support precision medicine. **Methods:** A narrative literature review was conducted using PubMed, Web of Science, and LILACS. The search strategy used controlled vocabulary terms indexed in Medical Subject Headings (MeSH) and Health Sciences Descriptors (DeCS), combined with Boolean operators. Studies addressing IBD epidemiology, pathophysiology, clinical manifestations, diagnosis, treatment, gut microbiota, microbial metabolites, and systemic complications were critically evaluated. **Results:** Current evidence indicates that dysbiosis and impaired intestinal barrier integrity are associated with alterations in microbial metabolites, including short-chain fatty acids, bile acids, lipids, and other microbiota-derived compounds. These alterations interact with immune and inflammatory pathways and may contribute to extraintestinal manifestations, particularly metabolic and hepatobiliary disorders through the gut–liver axis. Advances in metabolomics, multi-omics approaches, biologic agents, and targeted therapies are expanding opportunities for patient stratification and individualized treatment. **Conclusions:** IBD should be regarded as a systemic and multifactorial disorder involving interconnected microbial, metabolic, molecular, and immune mechanisms. Integrating these dimensions may improve disease monitoring, therapeutic selection, and the development of precision medicine strategies.

## 1. Introduction

Inflammatory bowel disease (IBD) is an umbrella term that primarily encompasses two chronic immune-mediated disorders, Crohn’s disease (CD) and ulcerative colitis (UC), both characterized by multifactorial pathogenesis and involvement of the gastrointestinal tract. These diseases follow a relapsing–remitting course, and their global burden has expanded beyond traditionally Westernized regions, making IBD a global public health concern because of its increasing incidence and prevalence, particularly in newly industrialized regions such as Latin America, Asia, and the Middle East [1,2].

IBD pathogenesis involves complex interactions among genetic susceptibility, epigenetic regulation, intestinal dysbiosis, epithelial barrier dysfunction, and dysregulated innate and adaptive immune responses [3,4]. Oxidative stress is an important component of IBD pathophysiology and results from an imbalance between the generation of reactive oxygen species (ROS) and cellular antioxidant capacity. This imbalance may promote epithelial injury and compromise intestinal barrier integrity [5].

Zhang et al. [6] demonstrated that deficiency of transcription factor EB (TFEB) in intestinal epithelial cells disrupts barrier function and increases susceptibility to experimental colitis. The authors also reported reduced TFEB expression in the colonic tissue of patients with IBD and identified the TFEB–peroxisome proliferator-activated receptor gamma coactivator 1-alpha (PGC-1α) axis as an important regulator of antioxidant homeostasis and ROS production, further supporting the mechanistic link between oxidative stress, epithelial integrity, and intestinal inflammation.

Epithelial ROS production also appears to be closely associated with intestinal inflammatory activity. Current evidence suggests that DUOX2 is an important epithelial source of ROS in IBD and that its expression is increased in inflamed intestinal mucosa. In patients with active ulcerative colitis, increased expression of DUOX2 and its maturation factor DUOXA2 has been associated with greater inflammatory activity and impaired epithelial barrier integrity. These findings support a mechanistic link between excessive epithelial ROS production, disruption of mucosal homeostasis, and sustained intestinal inflammation [7]. Collectively, dysregulated ROS production may contribute to the establishment of a pro-inflammatory intestinal microenvironment and perpetuate epithelial injury in IBD.

Persistent intestinal inflammation may extend beyond the gastrointestinal tract through systemic inflammatory pathways, contributing to extraintestinal manifestations involving the joints, skin, eyes, liver, cardiovascular system, bone metabolism, and neuropsychiatric axis [8,9,10].

In addition to its clinical impact, IBD represents a substantial socioeconomic burden because of recurrent hospitalizations, biologic therapies, surgical interventions, and reduced productivity. Therefore, understanding the molecular, cellular, and systemic mechanisms underlying IBD is essential for improving disease management and developing more personalized therapeutic strategies [11,12].

Although considerable progress has been made in understanding the pathogenesis of IBD, much of the current literature has addressed individual components of the disease separately, including genetic susceptibility, immune dysregulation, intestinal microbiota, and therapeutic advances. Little et al. [13] reported that ongoing developments in precision medicine are transforming the understanding of IBD by integrating molecular mechanisms with individualized approaches to disease prediction, monitoring, and therapeutic response. Similarly, Syed et al. [14] emphasized that future progress in IBD research will depend on integrating molecular biomarkers, multi-omics technologies, and detailed clinical phenotyping to improve patient stratification and optimize therapeutic decision-making. The originality of the present review lies in integrating these interconnected dimensions within a unified framework that links the multifactorial and metabolic mechanisms underlying intestinal inflammation, including epithelial barrier dysfunction, dysbiosis, oxidative stress, and genetic and epigenetic regulation, with the systemic and extraintestinal consequences of IBD. Rather than considering these mechanisms and clinical manifestations as isolated components, this review examines their interrelationships within the evolving concept of IBD as a systemic disorder.

Accordingly, this review provides an integrated and updated synthesis of current evidence on IBD pathophysiology, epidemiology, clinical and extraintestinal manifestations, diagnostic strategies, and therapeutic advances, with particular emphasis on the transition from a localized intestinal inflammatory disorder to a complex systemic disease. By connecting molecular, microbial, metabolic, and oxidative mechanisms with multisystem involvement and emerging precision medicine approaches, this review aims to provide a comprehensive framework for understanding IBD from its molecular basis to its multisystem clinical expression and to support increasingly individualized approaches to disease management.

## 2. Methods

### Literature Search Strategy

A narrative literature review was conducted to synthesize current evidence on IBD, with emphasis on epidemiology, pathophysiology, genetic and epigenetic mechanisms, intestinal microbiota, microbial and metabolic alterations, extraintestinal manifestations, diagnostic approaches, and therapeutic strategies.

The literature search was conducted in the PubMed, Web of Science, and LILACS databases and primarily included publications from 2012 to 2026. Earlier landmark studies were also considered when necessary to provide historical context or describe fundamental concepts and mechanisms relevant to IBD. The search strategy combined controlled descriptors indexed in Medical Subject Headings (MeSH) and Descritores em Ciências da Saúde (DeCS) with free-text terms related to IBD and the main topics addressed in this review. The following terms and their combinations were used: “Inflammatory Bowel Diseases,” “Crohn Disease,” “Ulcerative Colitis,” “intestinal microbiota,” “gut dysbiosis,” “microbial metabolites,” “genetics,” “epigenetics,” “extraintestinal manifestations,” “diagnosis,” “biomarkers,” “treatment,” and “precision medicine.” Search terms were combined using the Boolean operators AND and OR.

Studies were considered eligible if they addressed IBD, Crohn’s disease, or ulcerative colitis and provided information relevant to at least one of the topics predefined for this review. Based on their contribution to each topic, original observational and experimental studies, clinical trials, systematic reviews, meta-analyses, narrative reviews, clinical practice guidelines, and consensus statements published in peer-reviewed journals were considered for inclusion.

Studies were excluded if they were duplicates, were not directly related to IBD or the predefined scope of the review, focused on conditions unrelated to Crohn’s disease or ulcerative colitis without providing information relevant to IBD, or lacked sufficient methodological or scientific information to address the objectives of this review. Conference abstracts, editorials, letters to the editor, and other publications lacking sufficient scientific information for critical assessment were also excluded. In addition, the reference lists of selected articles were examined to identify relevant studies that had not been retrieved through the initial database searches.

Study selection was performed in two stages. First, titles and abstracts were screened for relevance to the scope of the review. Subsequently, potentially eligible articles underwent full-text assessment. The final selection considered thematic relevance, consistency with the scope of the review, and the scientific contribution of each publication to the topics addressed. The evidence was then synthesized narratively to provide an integrated and updated overview of the biological, microbial, metabolic, clinical, diagnostic, and therapeutic dimensions of IBD.

Because this study was designed as a narrative review, no formal risk-of-bias assessment or quantitative synthesis was performed. The methodological approach was intended to provide a comprehensive and critical synthesis of the available evidence, consistent with the principles described for narrative literature reviews [15].

## 3. Inflammatory Bowel Disease (IBD)

### 3.1. Definition

IBD comprises two major chronic inflammatory disorders, ulcerative colitis (UC) and Crohn’s disease (CD), both characterized by a multifactorial and incompletely understood etiology. Although classified within the same disease group, these disorders differ in genetic susceptibility, risk factors, and clinical, endoscopic, and histopathological characteristics [2]. Current evidence indicates that these disorders exhibit considerable immunological heterogeneity, reflecting complex interactions among genetic, environmental, microbial, epithelial, and immunological factors [16]. Among these mechanisms, distinct patterns of adaptive immune activation contribute to the pathophysiology of Crohn’s disease (CD) and ulcerative colitis (UC).

Ulcerative colitis is a chronic, idiopathic inflammatory condition affecting the colon and characterized by friability of the colonic mucosa and superficial erosions. It is frequently associated with hematochezia and other gastrointestinal manifestations. UC represents the most common form of IBD worldwide and follows a relapsing–remitting course, with disease extent ranging from the rectum to more proximal segments of the colon [17,18].

Crohn’s disease (CD), although also a chronic inflammatory condition, differs from UC in its discontinuous and transmural pattern of inflammation and its potential to affect any segment of the gastrointestinal tract. The colon and distal ileum are the most commonly involved sites; however, any region of the gastrointestinal tract, beginning in the oral cavity, may be affected [19]. Hallmark features of CD include discontinuous (“skip”) areas of inflammation characterized by extensive ulcerative lesions and edema and, in severe cases, luminal stenosis, granuloma formation, and transmural inflammation [20]. The lesion topography of both conditions is illustrated in Figure 1.

The pathogenesis of IBD is complex and involves interactions among genetic susceptibility, microenvironmental factors, environmental exposures, intestinal dysbiosis, metabolic alterations, epithelial barrier dysfunction, and dysregulated immune responses [3]. These interconnected mechanisms contribute to the initiation and perpetuation of chronic inflammation within the gastrointestinal tract (GIT) and are summarized in Figure 2.

The clinical expression of IBD is heterogeneous and varies according to disease phenotype, location, extent, and inflammatory activity. Patients with UC frequently present with rectal bleeding, diarrhea, increased bowel frequency, intestinal urgency, tenesmus, fecal incontinence, and abdominal cramping. In addition to intestinal manifestations, extraintestinal complications involving the joints, hepatobiliary system, and eyes may occur, including arthritis, cholangitis, cholecystitis, and ocular inflammation [21].

IBD also has important psychosocial consequences. Depression, anxiety, sleep disturbances, and sexual dysfunction have been reported, while the unpredictability of disease flares and the need for continuous treatment may substantially affect long-term life planning, social functioning, and financial stability [21]. Therefore, beyond understanding the biological mechanisms and epidemiological burden of IBD, it is essential to recognize its broader impact on patients’ daily lives and quality of life.

### 3.2. Epidemiology

Among the two major forms of IBD, ulcerative colitis (UC) generally has a higher prevalence than Crohn’s disease (CD). These conditions are most commonly diagnosed in adolescents and young adults, typically between 10 and 40 years of age. In CD, peak incidence is observed between 15 and 25 years of age [22]. Moreover, UC may exhibit a bimodal age distribution, with incidence peaks occurring during the second and eighth decades of life. Nevertheless, both conditions can affect individuals across a wide range of age groups [23].

Kaplan and Ng [24] emphasized that, although disease onset is most frequently observed between the second and fourth decades of life, the incidence of both pediatric-onset and older-onset IBD has increased considerably over recent decades. These demographic changes have introduced new clinical challenges related to diagnosis, therapeutic decision-making, and long-term disease management across different age groups.

Hracs et al. [1] proposed that the global epidemiology of IBD has entered a new stage characterized not only by increasing incidence in industrializing countries but also by rising prevalence in regions where the disease was previously considered uncommon. This epidemiological transition reflects the combined influence of urbanization, environmental changes, dietary westernization, and improvements in disease recognition and diagnostic capacity. These findings reinforce the growing importance of IBD as a global health challenge with substantial clinical and socioeconomic implications.

Over recent decades, the epidemiology of IBD has undergone substantial changes. Historically, the highest incidence and prevalence rates were reported in North America and Europe [25]. However, industrialization, urbanization, the adoption of Western lifestyles, and environmental changes have contributed to marked increases in disease incidence in newly industrialized regions. Consequently, Asia, the Middle East, Africa, and South America have emerged as regions experiencing a rapid increase in IBD incidence, reinforcing the important role of environmental factors in disease development [1,25,26].

Consistent with these observations, Kaplan and Windsor [27] characterized the current global expansion of IBD as a major epidemiological transition among chronic inflammatory diseases. According to the authors, countries undergoing rapid socioeconomic development are experiencing marked increases in IBD incidence, whereas industrialized countries have entered a phase in which incidence is stabilizing while prevalence continues to increase, largely because of improved survival, earlier diagnosis, and the cumulative growth of the affected population.

A summary of the principal epidemiological findings reported over the last five years, including incidence, prevalence, geographic distribution, study population, and study period, is presented in Table 1.

Building on these observations, Hracs et al. [1] proposed that the global evolution of IBD should no longer be interpreted exclusively in terms of incidence and prevalence rates, but also through distinct epidemiological stages that reflect demographic transitions, environmental exposures, healthcare infrastructure, and disease awareness across different populations. This updated framework provides a broader understanding of the current global burden of IBD and may support the development of region-specific prevention and healthcare strategies.

Kotze et al. [28] reported a progressive increase in the incidence and prevalence of IBD throughout Latin America and the Caribbean, although substantial differences remain among countries in terms of epidemiological data availability, disease phenotype, access to specialized care, and therapeutic resources. The authors also emphasized the need for additional population-based studies capable of accurately characterizing the evolving regional burden of IBD. These findings indicate that global epidemiological trends should be interpreted in the context of the socioeconomic, demographic, environmental, and healthcare characteristics of each region.

**Table 1:** Prevalence and Incidence of Inflammatory Bowel Disease (IBD).

Authors	Country	Period	Sample	Condition	Prevalence * (Rate and Unit as Reported)	Incidence * (Rate and Unit as Reported)
Crocetti [22]	Italy, Milan	2000–2018	15,141 prevalent; 3434 incident cases	IBD; UC; CD	2018 crude prevalence per 100,000 population: IBD 437.0; UC 271.9; CD 165.1 (M: 184.1; F: 147.2) 2010–2018 (standardized): UC 175.3 → 277.9; CD 107.1 → 171.8	2015–2018 crude incidence per 100,000 person-years: UC 15.6; CD 9.3 (M: 10.5; F: 8.2)
Renuzza [26]	Paraná, Brazil	2010–2019	6748 patients	IBD; Ulcerative Colitis (UC); Crohn’s Disease (CD)	IBD 17.19 → 58.88; UC 11.90 → 42.99; CD 5.29 → 15.84; mean: 2.76 → 10.74	IBD 2.00 → 13.77; UC 1.34 → 10.43; CD 0.66 → 3.34
Sarter [29]	France	1988 and 2017	22,879 patients	IBD; UC; CD; IBD unclassified (IBD-U)	—	IBD 12.7; UC 5.1; CD 7.2; IBD-U 0.37
Heydari [30]	Global	2000–2022	215 studies	IBD; UC; CD	IBD 229.7; UC 120.4; CD 84.2	IBD 9.7; UC 5.0; CD 4.0
Coward [31]	Canada (provinces)	Incidence: 2007–2014 Prevalence: 2002–2014	Representative data from >95% of the Canadian population	IBD; UC; CD	Prevalence per 100,000 population: IBD: 720 (Manitoba), 1239 (Nova Scotia), 843 (national) CD: 423 (2023) UC/IBD-U: 420 (2023)	Incidence per 100,000 population: IBD: 21.9 (Quebec), 44.7 (Newfoundland and Labrador), 41.6 (Nova Scotia) 2023: IBD 29.9; CD 12.7; UC/IBD-U 17.2
Wang [32]	Global	1990–2019	204 countries analyzed	IBD	3.32 → 4.90 million; age-standardized prevalence: 73.23 → 59.25/100,000 (1990–2019)	—
Seleq [33]	New Zealand, Waikato	2009 and 2019	Retrospective cohort study	IBD	IBD: 293.6 → 375.6 (2010–2019)	IBD: 21.5 → 17.5 (2010–2019)
Olivera [34]	Argentina, Buenos Aires	2018–2022	82 cases	IBD; UC; CD	IBD: 134; UC: 98; CD: 36	IBD: 11.93; CD: 2.88; UC: 9.05
Lin [35]	Global	1990–2021	204 countries analyzed	IBD	—	Age-standardized incidence rate: 4.22 → 4.45 per 100,000 population (1990–2021); incident cases increased by 88.30%.
Žulj [36]	Croatia, Osijek-Baranja	2000–2014	159 participants	IBD; UC; CD	UC: 3.0 → 109.5 per 100,000 population; CD: 2.5 → 63.3 per 100,000 population	517 incident IBD cases overall; UC: 71.4 → 30.5 per 100,000 population; CD: 37.5 → 21.0 per 100,000 population.
Nielsen [37]	Faroe Islands	1960 and 2020	873 patients	IBD; UC; CD; IBD-U	Prevalence per 100,000 persons: IBD 599 → 871 → 1414; UC 417 → 565 → 934; CD 96 → 159 → 248; IBD-U 86 → 147 → 232 (2001–2011–2021), 2020: Sandoy 1952; Eysturoy 1138	Incidence per 100,000 person-years: Sandoy: 0 → 74 → 106 (1960–2020) Neighboring islands: 14–27 → 41–63 (1980–2020) Eysturoy 63; Norðoyggjar 52; Southern Streymoy 41 (2010–2020)
Freeman [38]	United Kingdom	2006–2016	14 million patients	IBD	Prevalence per 10,000 adult population: IBD 106.2 → 142.1	Incidence per 100,000 person-years: IBD 76.4 (2010); 63.5 (2016); 69.5 overall
Dorn-Rasmussen [39]	Denmark	1980–2017	69,862 patients	IBD; UC; CD; IBD-U	Per 100,000 persons: IBD 58 → 893; CD 293 (2017); UC 523 (2017); IBD-U 77 (2017)	Per 100,000 person-years: IBD 44.4 (2017); UC 6.2 → 27.2; CD 5.1 → 15.6; IBD-U 1.2 → 7.2 → 1.6 (1980–2008–2017)
Kern et al. [40]	Germany, Saxony	2000–2014	532 patients	IBD; UC; CD; IBD-U	Prevalence per 100,000: IBD 111.8	Age-standardized incidence per 100,000 person-years: IBD 7.5; 4.6 → 8.2; CD 4.8; UC 2.5; IBD-U 0.3.

Note: * Prevalence is generally expressed as cases per 100,000 persons, whereas incidence is generally expressed as new cases per 100,000 population per year or per 100,000 person-years, according to the original study. When a different denominator or an absolute number of cases was reported, the corresponding unit is specified in the table.

### 3.3. Gut Microbiota, Microbial Metabolites, and Immune Dysregulation in IBD

The intestinal barrier represents a critical interface between the host and the external environment, maintaining intestinal homeostasis through continuous interactions with commensal microorganisms and their metabolites. The gut microbiota contributes to immune regulation, metabolic processes, digestion, defense mechanisms, and several other physiological functions of the host [41].

Current evidence supports the view that the intestinal barrier should be understood as a dynamic multicellular system composed not only of epithelial cells and intercellular junction proteins but also of the mucus layer, immune cells, vascular endothelium, enteric neurons, and the gut microbiota. Dunleavy et al. [42] reported that alterations in any of these interconnected components may disrupt mucosal homeostasis, thereby promoting bacterial translocation and perpetuating the chronic intestinal inflammation characteristic of IBD.

Loss of intestinal barrier integrity facilitates the translocation of microbial components and luminal antigens, thereby contributing to the maintenance of chronic inflammation. In IBD, alterations in mucus production, epithelial integrity, antimicrobial peptide secretion, and intercellular junction proteins, including occludins and claudins, increase intestinal permeability and facilitate immune activation within the lamina propria [42,43]. Furthermore, persistent epithelial injury may impair mucosal repair mechanisms, alter epithelial cell differentiation, and promote sustained activation of innate and adaptive immune responses [42].

At the mechanistic level, persistent inflammatory stress can impair epithelial cell bioenergetics and directly contribute to the loss of intestinal barrier integrity. In IBD, mitochondrial dysfunction is associated with impaired oxidative phosphorylation (OXPHOS) and reduced ATP-generating capacity, while metabolic reprogramming toward greater reliance on glycolysis has also been observed in the inflamed intestinal epithelium [44,45,46]. Increased epithelial expression of hexokinase 2 (HK2), a key glycolytic enzyme, has also been associated with the severity of intestinal inflammation in IBD, further supporting the involvement of altered cellular energy metabolism in the inflammatory process [47].

Mitochondrial dysfunction may also promote increased generation of mitochondrial reactive oxygen species (mtROS), thereby exacerbating oxidative stress and epithelial injury [45,48]. Excessive ROS production can damage cellular components essential for epithelial homeostasis, whereas reduced ATP availability may impair energy-dependent processes required for mucosal maintenance and repair. Thus, impaired OXPHOS, metabolic reprogramming, reduced ATP availability, and increased ROS production may act synergistically to disrupt epithelial homeostasis and compromise intestinal barrier integrity.

In healthy individuals, Firmicutes, Bacteroidetes, Proteobacteria, and Actinobacteria predominate within the gut microbiota, with Firmicutes and Bacteroidetes accounting for approximately 90% of its composition. These microorganisms contribute to intestinal homeostasis, particularly through the fermentation of dietary fibers and the production of short-chain fatty acids (SCFAs), such as butyrate and propionate, which play important roles in maintaining intestinal barrier integrity and regulating immune responses [49,50].

In contrast, dysbiosis in IBD is characterized by reduced microbial diversity, depletion of beneficial commensal microorganisms, and enrichment of potential pathobionts. Longitudinal studies have demonstrated greater variability and temporal instability of the gut microbiota in these patients, reinforcing the dynamic nature of IBD-associated dysbiosis [51,52]. Studies conducted in Indian populations have also identified microbiome alterations similar to those observed in Western populations [53].

Host genetic susceptibility may also influence gut microbiota composition. Yilmaz et al. [54] demonstrated that, in ulcerative colitis (UC), PTPN2 variants are associated with alterations involving Clostridiales, Lachnospiraceae, and Ruminococcus, accompanied by reduced *Roseburia* abundance. In severe Crohn’s disease (CD), reductions in Faecalibacterium, Bilophila, Coprococcus, and Erysipelotrichaceae were observed, whereas *Bacteroides* abundance was increased in individuals carrying PTPN2 WT. These findings further support the interaction between host genetic susceptibility, gut microbiota composition, and the inflammatory phenotype.

Several potentially pathogenic microorganisms have been associated with the initiation or exacerbation of intestinal inflammation, including *Salmonella*, *Shigella*, *Yersinia*, *Campylobacter*, *Aeromonas*, *Clostridioides difficile*, and pathogenic strains of *Escherichia coli* [55]. Jain and Venkataraman [53] and Lloyd-Price et al. [56] observed depletion of obligate anaerobes, including *Faecalibacterium prausnitzii* and *Roseburia hominis*, together with enrichment of facultative anaerobes such as *E. coli* in CD. Alterations involving *Ruminococcus torques* and *Ruminococcus gnavus* have also been implicated in IBD pathophysiology.

In CD, reductions in Christensenellaceae, Coriobacteriaceae, and *Faecalibacterium prausnitzii* have also been reported, accompanied by increased abundance of *Actinomyces*, *Veillonella*, and *Escherichia coli*. In UC, reductions in *Eubacterium rectale* and *Akkermansia* have been associated with increased *E. coli* abundance [43,44,57].

Collectively, these alterations indicate a shift from a diverse microbial community functionally associated with intestinal homeostasis toward a dysbiotic profile characterized by the loss of beneficial microorganisms and enrichment of microbial groups with pro-inflammatory potential. The main differences between the healthy gut microbiota and the microbiota associated with IBD are summarized in Figure 3.

Once established, dysbiosis can modulate both local and systemic immunity, establishing a bidirectional feedback loop between the microbiota and the immune system through microbial metabolites, epigenetic alterations, and changes in host gene expression. In this context, microbiota-derived metabolites such as propionic acid may exert immunomodulatory effects by regulating macrophage polarization and inflammatory signaling pathways, including the MAPK pathway [58]. At the same time, the inflammatory intestinal environment can modify the structure and function of microbial communities, thereby contributing to the perpetuation of inflammation [59].

Beyond taxonomic alterations, the contribution of the gut microbiota to IBD progression involves substantial changes in microbial metabolic activity. The depletion or expansion of specific microbial populations alters the production and biotransformation of metabolites capable of acting directly on epithelial and immune cells, thereby establishing an important mechanistic link between dysbiosis, intestinal barrier dysfunction, and inflammation [60]. Key microbiota-derived metabolites include SCFAs, tryptophan-derived metabolites, and secondary bile acids [61,62].

The depletion of SCFA-producing bacteria represents an important functional alteration associated with IBD. Beneficial bacteria, such as *Faecalibacterium prausnitzii* and *Roseburia* species, are major butyrate producers, and their depletion may compromise intestinal barrier integrity and immune regulation [63]. In addition to serving as an important energy source for colonocytes, butyrate exerts immunomodulatory effects through mechanisms involving G protein-coupled receptors (GPCRs) and inhibition of histone deacetylases (HDACs). Clinical studies have also shown that oral butyrate supplementation can modulate the gut microbiota and improve clinical and biochemical parameters in patients with IBD, further supporting its relevance to intestinal homeostasis [64].

Butyrate also participates in the regulation of regulatory T cells (Tregs) [62]. These effects are particularly relevant to the balance between Tregs and T helper 17 (Th17) cells. Microbiota-derived metabolites can modulate this balance through receptor-mediated signaling, epigenetic regulation, and metabolic reprogramming. Accordingly, reduced SCFA availability may impair immunoregulatory mechanisms and favor the persistence of mucosal inflammation [58,63,65].

Microbial tryptophan metabolism represents another important link between the gut microbiota and the immune response. Specific microorganisms convert tryptophan into indole and its derivatives, several of which act as ligands for the aryl hydrocarbon receptor (AhR). AhR-mediated signaling contributes to the maintenance of mucosal homeostasis, epithelial regeneration, and cytokine regulation, including IL-22, while also influencing Treg and Th17 responses [66]. Consequently, alterations in microbial tryptophan metabolism may disrupt epithelial and immune homeostasis and favor the persistence of intestinal inflammation [62,63].

Bile acid metabolism constitutes another important interface between the microbiota and the host. Primary bile acids undergo microbial biotransformation in the intestine, generating secondary bile acids, including lithocholic acid (LCA) and deoxycholic acid (DCA) [67]. These compounds act as signaling molecules capable of modulating epithelial, metabolic, and immune pathways. Alterations in this biotransformation may modify immunoregulatory signaling and influence the Treg/Th17 balance, thereby favoring a pro-inflammatory intestinal environment [62,63].

Integrated metabolomic and metagenomic analyses provide additional evidence supporting this functional relationship. Franzosa et al. [68] identified enrichment of sphingolipids and bile acids, together with reductions in triacylglycerols and tetrapyrroles, in patients with CD and UC. In addition, 122 robust associations between microbial species and metabolites were identified, demonstrating a close relationship between alterations in microbial composition and changes in intestinal metabolism. These findings reinforce the concept that dysbiosis in IBD should be interpreted not only in terms of taxonomic composition but also in terms of its functional and metabolic consequences.

The main relationships between alterations in the gut microbiota, microbial metabolites, and their potential consequences for IBD are summarized in Table 2.

The functional consequences of these alterations in the gut microbiota and microbial metabolites converge with the immune dysregulation characteristic of IBD.

In CD, evidence increasingly supports the involvement of Th1 and Th17 responses. Paroni et al. [80] identified a pathogenic intestinal population of CCR5+ Th17 cells associated with the inflamed mucosa of patients with CD. These cells exhibited a pro-inflammatory phenotype characterized by the production of IL-17 and IFN-γ, while IL-23 promoted their differentiation, further supporting the relevance of the IL-23/Th17 axis in sustaining intestinal inflammation.

Consistent with these findings, Lee et al. [81], using single-cell RNA sequencing combined with T-cell receptor sequencing, demonstrated an increased proportion of tissue-resident memory T cells with a Th17 signature in the small intestine of patients with CD. These cells exhibited increased expression of IL17A, IL22, CCR6, and CCL20, together with an inflammatory signature associated with IFN-γ. Leccese et al. [71] further demonstrated that adherent-invasive *E. coli* (AIEC) can promote the transdifferentiation of conventional Th17 cells into pathogenic IFN-γ-producing Th17 cells through mechanisms involving IL-23, further supporting the interaction between microbial stimuli and pathogenic immune responses in CD.

In UC, T-cell-mediated inflammation also involves alterations in the balance between effector and regulatory responses, particularly between Th17 and regulatory T (Treg) cells. Current evidence supports a more heterogeneous immunological profile than that suggested by the traditional classification of UC as a predominantly Th2-mediated response. Mediators such as TNF-α, IL-17, IL-23, IL-1β, and IL-6 contribute to the inflammatory microenvironment and influence the differentiation and activity of T-cell populations [71,81].

In this context, Wang et al. [82] demonstrated, using an experimental human cell model relevant to UC, that activation of the mTOR/HIF-1α pathway promotes Th17-cell differentiation, reduces the proportion of Treg cells, and increases the production of pro-inflammatory mediators. In contrast, mTOR silencing reduced Th17 differentiation and the levels of pro-inflammatory mediators while favoring anti-inflammatory responses. These findings support the involvement of Th17/Treg imbalance in the persistence of intestinal inflammation. Collectively, CD and UC involve complex and partially overlapping networks of T cells and cytokines and therefore cannot be adequately explained by a rigid dichotomy between Th1- and Th2-mediated responses.

The development of high-throughput sequencing technologies and multi-omics approaches has substantially expanded our understanding of these interactions. Iliev et al. [41] emphasized that alterations in microbial metabolic activity, host–microbiota interactions, and microorganism-derived signaling molecules play important roles in the initiation and progression of IBD and in therapeutic responses, supporting a shift from purely descriptive analyses of the microbiome toward its functional characterization.

Multi-omics approaches integrate complementary molecular layers, including genomics, transcriptomics, proteomics, metagenomics, metatranscriptomics, and metabolomics. This integration enables the simultaneous characterization of host genetic susceptibility, microbiota composition and function, microbial metabolic activity, and molecular alterations associated with the immune response. Metagenomic and metabolomic analyses have identified alterations in lipid metabolism, bile acids, SCFAs, and other microbiota-derived metabolites associated with disease activity [68].

In a large-scale longitudinal multi-omics analysis, Lloyd-Price et al. [56] demonstrated coordinated alterations in microbiota composition and transcriptional activity, accompanied by changes in the metabolic profiles of patients with IBD. These findings illustrate the dynamic interplay between the gut microbiome and the host.

Metabolomics is particularly relevant in this context because microbial metabolites can directly influence epithelial integrity and immune responses. Bhosle et al. [83] identified more than 1000 potentially bioactive metabolic features associated with IBD, including bile acids, SCFAs, bilirubin derivatives, polyamines, and vitamins.

Evidence derived from multi-omics approaches demonstrates that microbiota composition and function, microbial metabolism, epithelial barrier integrity, and host immune responses constitute interdependent components of IBD pathophysiology. The integration of these molecular and cellular layers and their relationship with disease progression are summarized in Figure 4.

Collectively, this evidence demonstrates that IBD progression results from a dynamic interplay among dysbiosis, alterations in microbial metabolism, disruption of intestinal barrier integrity, and dysregulation of immune responses [84]. Multi-omics approaches may contribute to the identification of biomarkers, patient stratification, and the development of more individualized therapeutic strategies. In addition, dietary factors, including a high intake of saturated fats, ultra-processed foods, and food additives, may promote dysbiosis and activate inflammatory pathways, thereby influencing both susceptibility to and progression of IBD [85].

### 3.4. Genetics and Epigenetics

Genetic sequencing is considered the gold standard for detecting and comprehensively characterizing genomic variations in IBD [86]. The contribution of genetic factors to the etiology of IBD is supported by evidence showing that approximately 5% to 23% of patients have an affected first-degree relative [3].

Although more than 250 susceptibility loci have been associated with IBD, Jans and Cleynen [87] emphasized that most genetic variants individually confer only modest effects on disease risk. Current evidence indicates that disease susceptibility results from the cumulative interaction of multiple genetic variants with environmental exposures, intestinal microbiota, and immune regulatory pathways, rather than from alterations in a single gene.

According to Jarmakiewicz-Czaja et al. [10], ATG16L1 T300A, CARD9, and CLEC7A show strong associations with IBD. The first gene linked to IBD was NOD2, which encodes nucleotide-binding oligomerization domain-containing protein 2. NOD2 is an intracellular receptor for microbial components that is expressed in intestinal immune cells and is involved in autophagy and the induction of inflammatory responses.

The three main NOD2 variants, R702W (Arg702Trp), G908R (Gly908Arg), and L1007fsinsC, are associated with Crohn’s disease [88]. In a bibliographic analysis conducted by Garza-Hernandez, Estrada, and Treviño [89], 126 genes were found to have experimental evidence of association with Crohn’s disease, including NOD2, TNF, IL23R, ATG16L1, TLR4, IL10, SLC22A4, SLC22A5, and IRGM.

Beyond the identification of susceptibility genes, studies have demonstrated that many IBD-associated variants converge on common biological pathways involving epithelial barrier integrity, autophagy, innate immune sensing, cytokine signaling, and host–microbiota interactions. Jans and Cleynen [87] proposed that understanding these shared molecular pathways may provide greater biological insight than evaluating isolated genetic variants.

ATG16L1 and NOD2 play key roles in maintaining epithelial homeostasis, suppressing inflammatory responses, regulating pro-inflammatory cytokine production, and promoting granule exocytosis by Paneth cells, processes that are crucial for intestinal barrier defense [90].

CARD9 deficiency in mice alters epithelial cell turnover and impairs colonic wound healing. It is also associated with defective expression of IL-22-induced antimicrobial peptides and reduced levels of Reg3g, Reg3b, and Il17a, which are involved in mucosal recovery and colonic defense. In addition, CARD9 deficiency promotes fungal dysbiosis. Furthermore, CARD9 deficiency disrupts microbial tryptophan metabolism, leading to reduced production of aryl hydrocarbon receptor ligands and exacerbation of intestinal inflammation [91].

CLEC7A and CLEC5A genes are also associated with susceptibility to IBD. The AA genotype of the rs2078178 polymorphism and the CT genotype of rs16910631 were significantly more frequent in patients with ulcerative colitis than in healthy individuals. The rs1285933 polymorphism in CLEC5A demonstrated a protective effect, as the GA genotype was associated with a reduced risk of both UC and CD. Additionally, the AA genotype conferred further protection against CD, and the A allele of this polymorphism was associated with reduced susceptibility to CD [92]. To date, approximately 240 risk loci associated with IBD have been identified [93].

In families with multiple affected members, the shared genetic burden is high. As demonstrated by Jans and Cleynen [87], individuals with a high polygenic risk score tend to develop IBD, whereas unaffected individuals typically exhibit low scores, indicating that this factor alone is not sufficient to determine disease manifestation. This finding reinforces the concept that familial IBD is not genetically uniform.

Epigenetic alterations play a fundamental role in the pathophysiology of IBD. The main mechanisms include DNA methylation, post-translational histone modifications, such as acetylation and methylation, and regulation by non-coding RNAs, particularly microRNAs (miRNAs) [94]. Jones et al. [95] reported high mRNA expression of Mbd2 in the distal colon of mice. Mbd2 functions as an epigenetic regulator linking DNA methylation to transcriptional repression and plays a central role in inflammation. Reduced levels of Mbd2 in IBD are associated with colon shortening, weight loss, and histological alterations. Another study demonstrated epigenetic silencing of RASSF1A, a tumor suppressor gene, in the descending colon of patients with IBD; this silencing may serve as a predictive factor for colorectal cancer in IBD [96].

In this context, Noble et al. [94] reported that genetic susceptibility alone is insufficient to explain disease onset and progression. Instead, epigenetic mechanisms function as dynamic regulators capable of integrating environmental exposures, microbial signals, dietary factors, and inflammatory stimuli, thereby influencing gene expression throughout the course of IBD. This concept may help explain the marked heterogeneity observed in clinical phenotype and therapeutic response among genetically susceptible individuals.

According to Howell et al. [97], in Crohn’s disease, epithelial changes occur in the terminal ileum, whereas in the colon, both CD and UC exhibit distinct patterns of methylation and transcription. These epigenetic alterations remain stable over time, suggesting the presence of a potential epithelial memory.

During acute intestinal inflammation, the gut microbiota can induce extensive epigenetic reprogramming in host intestinal cells, affecting regulatory pathways associated with colitis and colorectal cancer. Genetic intervention studies have further demonstrated that this microbiota-induced epigenetic programming of host cells plays an important role in maintaining intestinal homeostasis in vivo [98].

Research on epigenetic regulation has substantially expanded our understanding of IBD pathophysiology and its therapeutic implications. Accordingly, epigenetic mechanisms may represent promising targets for the development of novel targeted therapies [4].

Advances in genomics, epigenomics, transcriptomics, and single-cell sequencing are progressively improving our understanding of IBD pathogenesis and supporting the development of precision medicine strategies. Noble et al. [94] emphasized that integrating multi-omics data with clinical and environmental information may facilitate more accurate disease stratification, individualized therapeutic selection, and prediction of treatment response in future clinical practice. The main genetic and epigenetic mechanisms contributing to IBD pathogenesis, together with their interactions with environmental factors, gut microbiota, epithelial homeostasis, and immune regulation, are summarized in Figure 5.

### 3.5. Extraintestinal Manifestations (EIMs)

EIMs are frequent in patients with IBD and can affect multiple organs, particularly the joints, skin, and eyes. A systematic review and meta-analysis estimated an overall prevalence of approximately 24%, with a higher frequency among patients with Crohn’s disease than among those with ulcerative colitis. Ocular manifestations, peripheral arthritis, and erythema nodosum are more strongly associated with CD, whereas axial involvement and pyoderma gangrenosum may occur in both conditions [99].

Current understanding supports the view that extraintestinal manifestations should be regarded as integral components of IBD rather than secondary complications. Gordon et al. [9] emphasized that these manifestations frequently share common pathogenic mechanisms with intestinal inflammation, including dysregulated immune responses, genetic susceptibility, and systemic cytokine activation. In some patients, extraintestinal manifestations may precede gastrointestinal symptoms, contributing to diagnostic delay and reinforcing the systemic nature of IBD.

Patients with IBD also have an increased risk of systemic complications, including venous thromboembolism, cardiovascular diseases, and hematological abnormalities. Venous thromboembolic events occur more frequently in patients with IBD than in the general population and affect both patients with CD and those with UC [100]. Additionally, IBD has been associated with increased risks of stroke and atrial fibrillation [101,102]. Anemia remains one of the most common extraintestinal complications, contributing significantly to reduced quality of life and overall disease burden [103].

Chronic systemic inflammation also contributes to endothelial dysfunction, accelerated atherosclerosis, and alterations in coagulation pathways. Gordon et al. [9] noted that controlling intestinal inflammation is one of the most effective strategies for reducing the risk of several extraintestinal complications, particularly thromboembolic and cardiovascular events.

Metabolic and hepatic alterations are increasingly recognized in IBD. Patients with IBD may have an increased risk of metabolic syndrome, diabetes mellitus, and metabolic dysfunction-associated steatotic liver disease (MASLD), which are associated with chronic inflammation, intestinal dysbiosis, increased intestinal permeability, and metabolic disturbances [104,105,106,107,108]. Hepatobiliary complications include steatotic liver disease, cholelithiasis, and primary sclerosing cholangitis, the latter representing one of the most severe liver-associated manifestations [105,106,107].

Growing evidence supports the concept of the gut–liver axis as a bidirectional communication network linking intestinal inflammation with hepatobiliary disease. Hsu and Schnabl [109] demonstrated that disruption of the intestinal epithelial barrier promotes bacterial translocation, microbial metabolite dysregulation, and activation of hepatic immune responses, providing mechanistic support for the development of hepatobiliary disorders frequently associated with IBD, including primary sclerosing cholangitis and steatotic liver disease. These findings reinforce the importance of intestinal barrier integrity in maintaining both intestinal and hepatic homeostasis.

Musculoskeletal involvement is among the most prevalent EIMs and includes peripheral arthritis, axial spondyloarthritis, and enthesitis, with a higher frequency reported in patients with CD. In addition, reduced bone mineral density and osteoporosis are associated with disease severity, low body mass index, nutritional deficiencies, and corticosteroid exposure [110,111].

Ocular manifestations, including episcleritis, scleritis, and uveitis, occur more frequently in CD and represent important complications requiring multidisciplinary management [112]. Renal complications, although less frequent, may also occur and require early recognition to prevent progression [113]. Cutaneous manifestations include erythema nodosum, pyoderma gangrenosum, Sweet’s syndrome, aphthous ulcers, and drug-related skin reactions [9].

In addition to organ-specific complications, IBD is associated with a substantial psychological burden, including anxiety, depression, and social impairment, which may negatively influence disease activity and quality of life [114]. Therefore, the systemic nature of IBD reinforces the need for multidisciplinary approaches to ensure comprehensive patient management [8,9].

Gordon et al. [9] emphasized that the recognition of extraintestinal manifestations has important therapeutic implications because their presence frequently influences treatment selection, multidisciplinary follow-up, and long-term prognosis. Early identification and coordinated management involving gastroenterologists, rheumatologists, dermatologists, hepatologists, ophthalmologists, and mental health professionals are essential to optimize disease control and improve patients’ quality of life. These systemic and extraintestinal manifestations are summarized in Figure 6.

### 3.6. Clinical and Phenotypic Classification of Inflammatory Bowel Disease

Accurate clinical classification of IBD is essential for guiding individualized therapeutic strategies and informing prognosis. In ulcerative colitis, diagnosis relies on the integration of clinical manifestations, endoscopic assessment, and histopathological findings. The disease is characterized by continuous mucosal inflammation that typically originates in the rectum and extends proximally to a variable extent. In some patients with proctitis or left-sided colitis, a periappendiceal inflammatory lesion, known as a cecal patch, may also be observed. Disease severity is primarily determined by clinical parameters, including stool frequency and the presence of systemic inflammatory manifestations [115]. The criteria used for this stratification are presented in Table 3.

Crohn’s disease is classified according to age at diagnosis, disease location, and disease behavior. The Montreal Classification, derived from the Vienna Classification, provides the current framework for phenotypic characterization, as summarized in Table 4. Disease activity is commonly evaluated using the Crohn’s Disease Activity Index (CDAI), a validated composite score that integrates multiple clinical variables to quantify disease severity and monitor clinical progression [20,117], as shown in Table 5.

Moran et al. [118] emphasized that the Montreal Classification remains the international standard for phenotypic characterization of IBD because it provides a structured framework for describing disease location, behavior, and age at diagnosis. However, the authors also highlighted that the disease phenotype is dynamic rather than static and may evolve throughout the clinical course, reinforcing the importance of periodic reassessment during patient follow-up.

**Table 4:** Vienna and Montreal Classifications for Crohn’s Disease According to Age at Diagnosis, Disease Location, and Behavior (adapted from Lusetti [119]).

Category	Subtype	Vienna	Montreal
Age at diagnosis	A1	below 40 years	below 16 years
	A2	above 40 years	17–40 years
	A3	—	above 40 years
Disease location	L1	ileum	ileum
	L2	colon	colon
	L3	—	ileocolonic
	L4	upper gastrointestinal tract	isolated upper gastrointestinal disease *
Disease behavior	B1	non-stricturing, non-penetrating	non-stricturing, non-penetrating
	B2	stricturing	stricturing
	B3	penetrating	penetrating
	p	—	perianal disease modifier

Note: * In the Montreal classification, L4 indicates upper gastrointestinal involvement and may coexist with L1, L2, or L3 when upper gastrointestinal disease occurs together with ileal, colonic, or ileocolonic involvement.

**Table 5:** Crohn’s Disease Activity Index (CDAI) (adapted from Imbrizi et al. [20]).

Component	Description/Criteria	Score	Weighting Factor
Liquid or soft stools	Total number over the last 7 days	Absolute value	×2
Abdominal pain	None (0); Mild (1); Moderate (2); Severe (3)—weekly sum	0–21	×5
General well-being	Excellent (0); Good (1); Fair (2); Poor (3); Very poor (4)—weekly sum	0–28	×7
Associated symptoms/complications	Arthralgia/arthritis; iritis/uveitis; erythema nodosum; oral aphthae; anal fissure; fistulas/abscesses; other fistulas; fever	1 point per item (max. 6)	×20
Use of antidiarrheal drugs	No (0); Yes (1)	0–1	×30
Abdominal mass	None (0); Questionable (2); Definite (5)	0–5	×10
Hematocrit deviation	Males: 47 − Hct; Females: 42 − Hct; subtract rather than add if the patient’s Hct is above the reference value	Absolute deviation	×6
Body weight	Percentage deviation from standard weight *	%	×1
CDAI Interpretation	**Total Score/Classification**		
≤150	Remission		
151–250	Mild disease		
251–350	Moderate disease		
>350	Severe disease		

Note: * Standard (expected or ideal) weight is calculated as height (m)^2^ × 25.5 for males and height (m)^2^ × 22.5 for females. If the patient’s actual weight exceeds the expected weight, the resulting value is subtracted rather than added to the CDAI score.

In UC, the Mayo Score remains one of the most widely used instruments for assessing inflammatory activity and therapeutic response. This index integrates clinical and endoscopic parameters, including stool frequency, rectal bleeding, endoscopic appearance, and the physician’s global assessment. The total score ranges from 0 to 12, allowing for stratification of disease activity into remission, mild, moderate, or severe categories. Additionally, the endoscopic subscore provides an objective measure of mucosal inflammatory severity and is an important parameter for evaluating endoscopic response to therapy [120,121], as shown in Table 6.

Annese and Annese [122] proposed that future disease classification should extend beyond conventional clinical characteristics by integrating molecular biomarkers, genetic susceptibility, transcriptomic signatures, microbiome profiles, and imaging findings. According to the authors, combining these complementary data may improve patient stratification and facilitate individualized therapeutic decision-making, supporting the transition toward precision medicine in IBD.

In pediatric populations, validated disease-specific instruments are used for the standardized assessment of IBD activity. The Pediatric Crohn’s Disease Activity Index (PCDAI) incorporates clinical, laboratory, nutritional, and growth-related parameters to evaluate CD severity, whereas the Pediatric Ulcerative Colitis Activity Index (PUCAI) assesses UC activity based exclusively on clinical criteria, enabling disease monitoring without the need for routine endoscopic evaluation [123,124].

### 3.7. Diagnosis and Treatment

The diagnosis of IBD relies on an integrated evaluation of clinical manifestations, laboratory biomarkers, endoscopic findings, histopathological examination, and imaging techniques. Clinical presentation is heterogeneous and may include chronic diarrhea, abdominal pain, rectal bleeding, urgency, weight loss, fatigue, anemia, and growth impairment in pediatric patients. In both CD and UC, inflammatory alterations may, in some cases, precede overt clinical manifestations, potentially contributing to diagnostic delay [117,125,126].

Kucharzik et al. [127] emphasized that no single diagnostic modality is sufficient to establish a diagnosis of IBD. Instead, diagnosis requires the integration of clinical presentation, laboratory biomarkers, endoscopic findings, histopathology, and cross-sectional imaging, together with the exclusion of infectious, ischemic, and other inflammatory conditions that may mimic IBD. This multidisciplinary approach improves diagnostic accuracy and facilitates early disease characterization.

Laboratory evaluation includes systemic inflammatory markers such as C-reactive protein (CRP) and erythrocyte sedimentation rate (ESR), as well as fecal biomarkers, including calprotectin, lactoferrin, and S100A12, which are useful for assessing intestinal inflammation and monitoring disease activity [128].

Ileocolonoscopy with histological assessment remains central to diagnosis, allowing for evaluation of disease extent, differentiation between CD and UC, and assessment of mucosal damage. Cross-sectional imaging, including magnetic resonance enterography (MRE) and computed tomography enterography (CTE), complements endoscopic evaluation, particularly for detecting small-bowel involvement and complications in CD [129,130].

Costa et al. [131] emphasized that fecal calprotectin has become one of the most valuable non-invasive biomarkers for evaluating intestinal inflammation. In addition to supporting the differential diagnosis between IBD and functional gastrointestinal disorders, serial measurements of fecal calprotectin are increasingly used to assess mucosal healing, monitor disease activity, predict relapse, and evaluate therapeutic response.

Macroscopically, CD is characterized by discontinuous inflammation, transmural involvement, and skip lesions, whereas UC typically presents with continuous inflammation beginning in the rectum and extending proximally. Histological assessment identifies chronic inflammatory changes, including crypt architectural distortion, basal plasmacytosis, cryptitis, crypt abscesses, epithelial damage, and inflammatory infiltration of the lamina propria, which are important for diagnosis and disease classification [132,133,134].

Treatment strategies for IBD are individualized according to disease severity, location, behavior, previous therapeutic exposure, comorbidities, and patient-specific characteristics. Conventional therapies include aminosalicylates, corticosteroids, and immunomodulators. Aminosalicylates are primarily used for the induction and maintenance of remission in mild-to-moderate UC, whereas their therapeutic role in CD is limited. Corticosteroids are effective for inducing remission in active disease but are not recommended for long-term maintenance because prolonged exposure is associated with substantial adverse effects. Immunomodulators, including thiopurines and methotrexate, may be used as steroid-sparing or maintenance strategies in selected patients [135,136,137].

Advanced therapies have substantially transformed IBD management and include anti-tumor necrosis factor (anti-TNF) agents, anti-integrin therapies, agents targeting the IL-12/23 pathway or selectively targeting IL-23, and oral small molecules such as Janus kinase (JAK) inhibitors and sphingosine-1-phosphate (S1P) receptor modulators. These therapies have expanded the possibilities for achieving clinical and endoscopic remission, particularly in patients with moderate-to-severe disease or an inadequate response to conventional treatment [138,139,140]. Additional investigational approaches, including peptide-based therapeutic strategies and other emerging molecular targets, may further contribute to the development of more precise and individualized treatment options [141,142].

Overall, progress in diagnostic tools and therapeutic strategies has enabled a more tailored approach to IBD management, with the goals of achieving sustained remission, reducing complications, and improving long-term patient outcomes. In this context, Little et al. [13] emphasized the growing importance of integrating molecular and clinical information to support individualized therapeutic decision-making and precision medicine in IBD. Comparative assessment of therapeutic class, clinical efficacy, loss-of-response patterns, safety profiles, and treatment persistence may further support therapeutic positioning according to individual patient and disease characteristics. These parameters are summarized in Table 7, providing a clinically relevant framework for treatment selection and precision medicine in IBD.

### 3.8. Future Perspectives

Prospective studies indicate that the global burden of IBD will continue to increase in the coming decades, representing a major public health challenge. Zhang et al. [162] projected substantial increases in IBD prevalence in Hong Kong, Japan, and the United States, with a greater burden among younger populations in the latter two countries. These epidemiological trends reinforce the need for more effective, individualized, and sustainable strategies for disease prevention, diagnosis, monitoring, and treatment.

Globally, although incidence rates have stabilized or slightly declined in several high-income countries, prevalence continues to rise because of population aging and ongoing disease incidence in emerging regions [1,31]. Pediatric-onset IBD is expected to become increasingly relevant, although epidemiological data remain limited in developing countries [163]. Rapid socioeconomic changes, urbanization, dietary transitions, and environmental exposures have contributed to the increasing incidence observed in regions such as China and Latin America [164,165].

Strengthening epidemiological surveillance systems and incorporating environmental, genetic, and clinical risk factors into predictive models will be essential for healthcare planning and resource allocation, particularly in regions with limited healthcare infrastructure [32,166]. Furthermore, reducing the economic burden of IBD will require strategies that improve access to effective therapies, optimize healthcare costs, and minimize productivity losses associated with the disease [167].

Little et al. [13] proposed that the future management of IBD is likely to transition from a one-size-fits-all approach toward precision medicine. Integrating clinical characteristics with genetic susceptibility, transcriptomic and proteomic signatures, microbiome composition, and other multi-omics data may improve patient stratification, predict therapeutic response, and facilitate individualized treatment strategies.

Honap et al. [168] further suggested that future advances in IBD management will depend on integrating innovative therapeutic agents with predictive biomarkers capable of identifying patients most likely to benefit from specific treatments. This integrated approach may accelerate the implementation of precision medicine while improving therapeutic efficacy and reducing unnecessary drug exposure.

Natural products and advanced drug delivery systems also represent promising future strategies for IBD. Feng et al. [169] described the therapeutic potential of natural bioactive compounds in IBD, while Weng et al. [170] emphasized that nanoparticle-based delivery systems may improve controlled drug release and intestinal targeting. However, further clinical validation is required before these approaches can be widely incorporated into IBD treatment.

Emerging digital technologies are also expected to transform IBD management. Silverman et al. [171] identified several promising applications of artificial intelligence, including endoscopic assessment, prediction of therapeutic response, disease monitoring, clinical decision support, and clinical trial design. As these technologies continue to evolve, they may improve personalized care and optimize long-term clinical outcomes, although further prospective validation remains necessary.

Collectively, these developments are expected to transform IBD management from a reactive model toward a more predictive, preventive, and personalized approach.

## Figures and Tables

**Figure 1:**
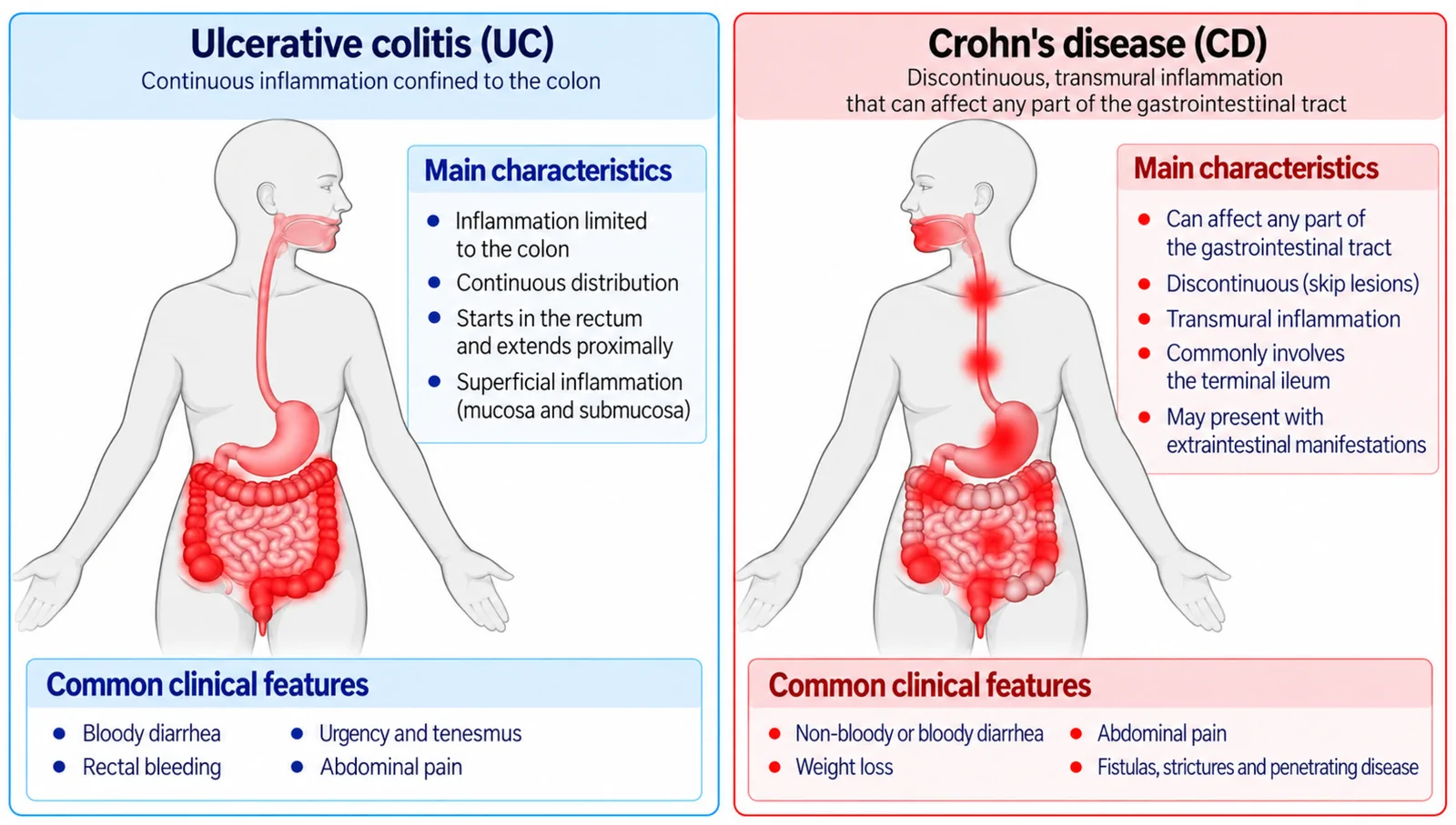
Topographic distribution of lesions in ulcerative colitis and Crohn’s disease. Comparative schematic representation of the gastrointestinal tract illustrating the distinct patterns of involvement in ulcerative colitis (UC) and Crohn’s disease (CD). Ulcerative colitis is restricted to the colon and rectum and is characterized by continuous mucosal inflammation. In contrast, Crohn’s disease may involve any segment of the gastrointestinal tract and is typically characterized by discontinuous (“skip”) lesions and transmural inflammation. The figure was created using BioRender resources (BioRender.com), accessed on 15 August 2026, with visual refinement for clarity and conceptual and visual assistance from ChatGPT (GPT-6, OpenAI, 2026).

**Figure 2:**
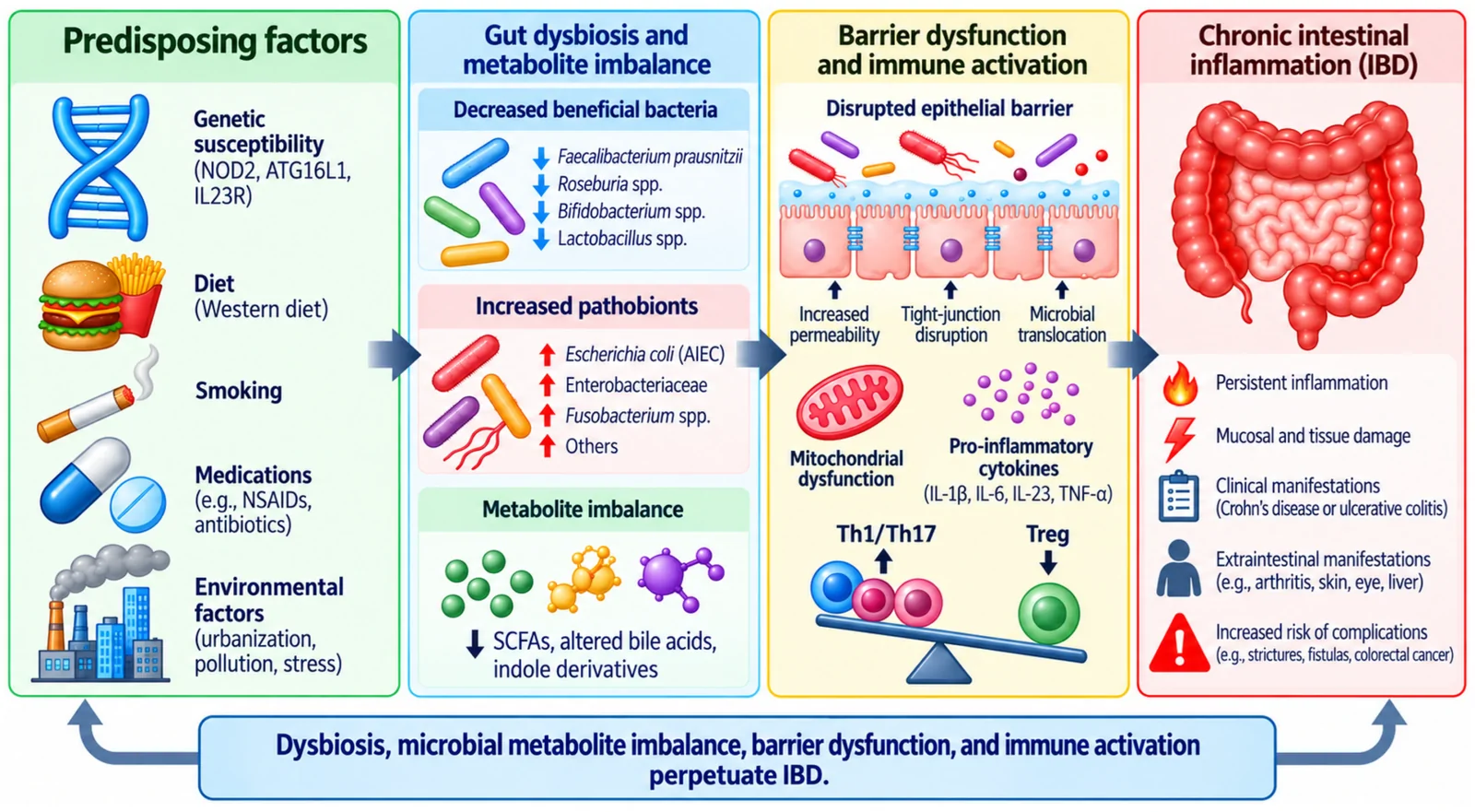
Integrated overview of the multifactorial pathogenesis of inflammatory bowel disease (IBD). Schematic representation of the main mechanisms involved in IBD pathogenesis, highlighting the interactions among genetic susceptibility, environmental factors, gut dysbiosis, microbial metabolite imbalance, epithelial barrier dysfunction, and immune activation. These interconnected alterations contribute to increased intestinal permeability, mitochondrial dysfunction, dysregulated inflammatory responses, and persistent intestinal inflammation, ultimately promoting tissue damage, extraintestinal manifestations, and disease-related complications. The figure was created using BioRender resources (BioRender.com), accessed on 13 September 2026, with visual refinement for clarity and conceptual and visual assistance from ChatGPT (GPT-6, OpenAI, 2026).

**Figure 3:**
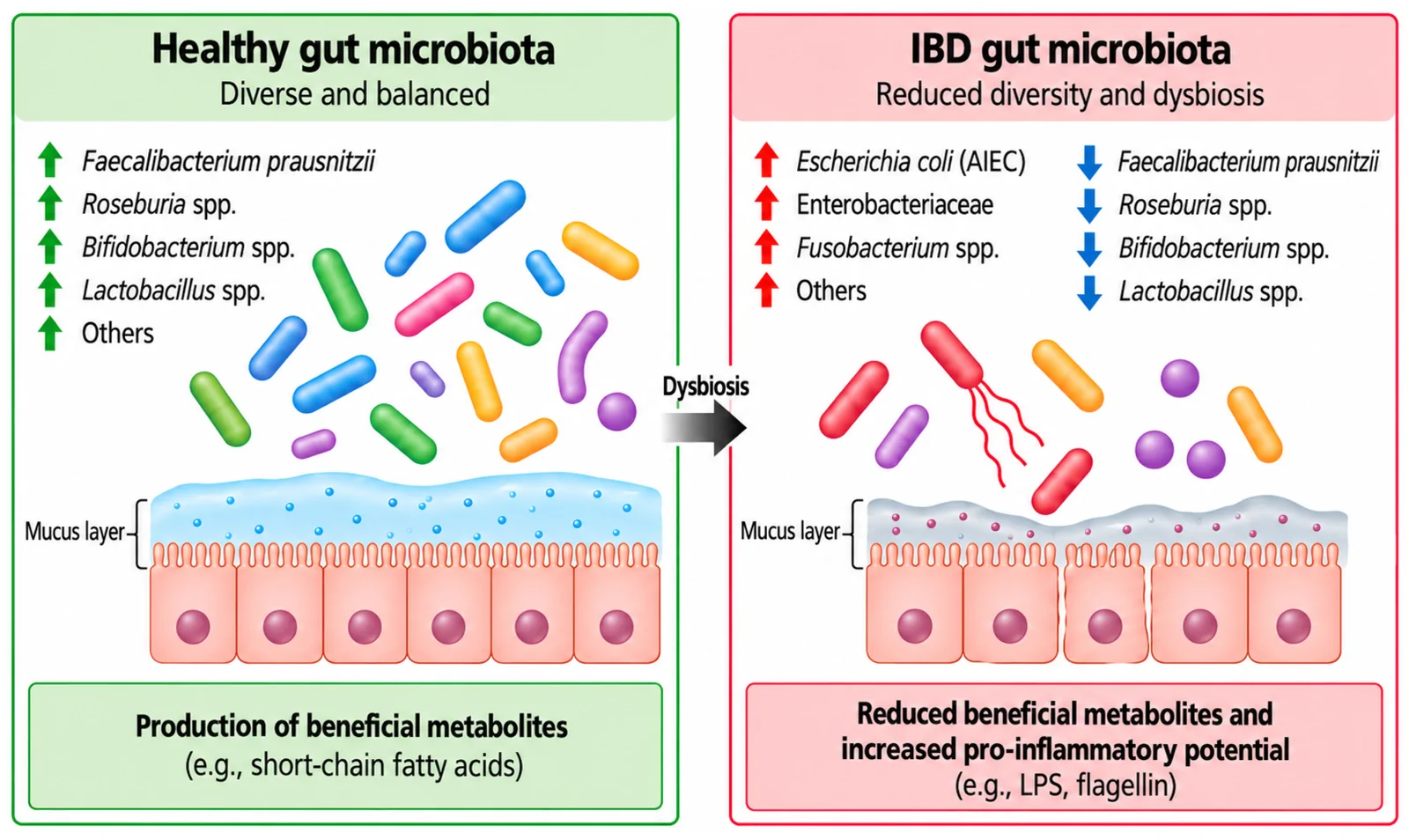
Alterations in gut microbiota associated with inflammatory bowel disease (IBD). Schematic representation of IBD-associated dysbiosis, characterized by reduced microbial diversity, depletion of beneficial bacteria, and enrichment of potential pathobionts. These alterations are associated with changes in microbial metabolite production, disruption of the intestinal barrier, and increased pro-inflammatory activity, thereby contributing to the persistence of intestinal inflammation. The figure was created using BioRender resources (BioRender.com), accessed on 10 September 2026, with visual refinement for clarity and conceptual and visual assistance from ChatGPT (GPT-6, OpenAI, 2026).

**Figure 4:**
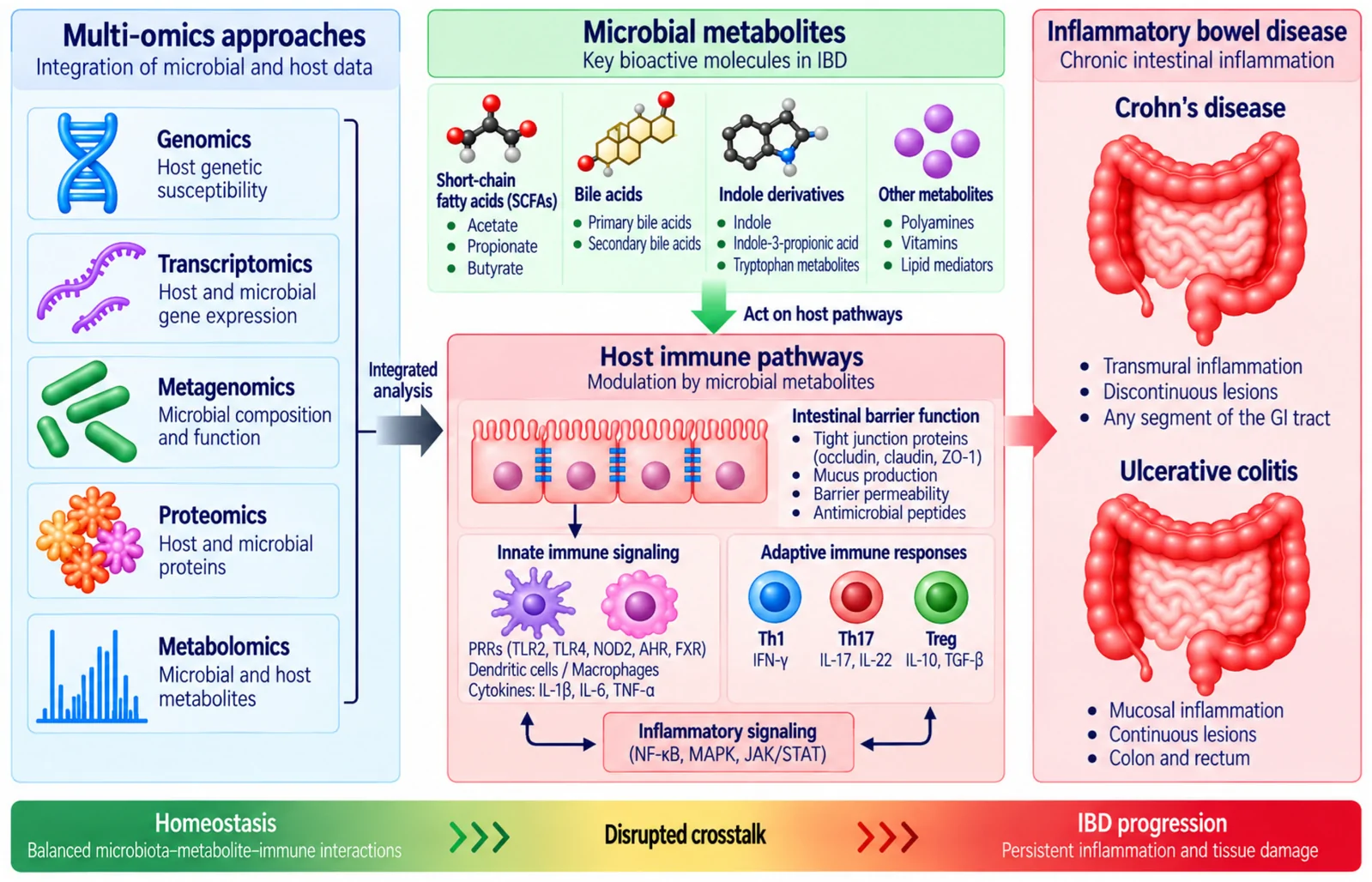
Role of gut dysbiosis and microbial metabolites in the development of inflammatory bowel disease (IBD). Conceptual overview of the interactions between gut dysbiosis, microbial metabolite alterations, intestinal barrier dysfunction, and immune activation. These changes promote increased intestinal permeability and pro-inflammatory responses, thereby contributing to persistent intestinal inflammation and the development and progression of Crohn’s disease and ulcerative colitis. The figure was created using BioRender resources (BioRender.com), accessed on 10 September 2026, with visual refinement for clarity and conceptual and visual assistance from ChatGPT (GPT-6, OpenAI, 2026).

**Figure 5:**
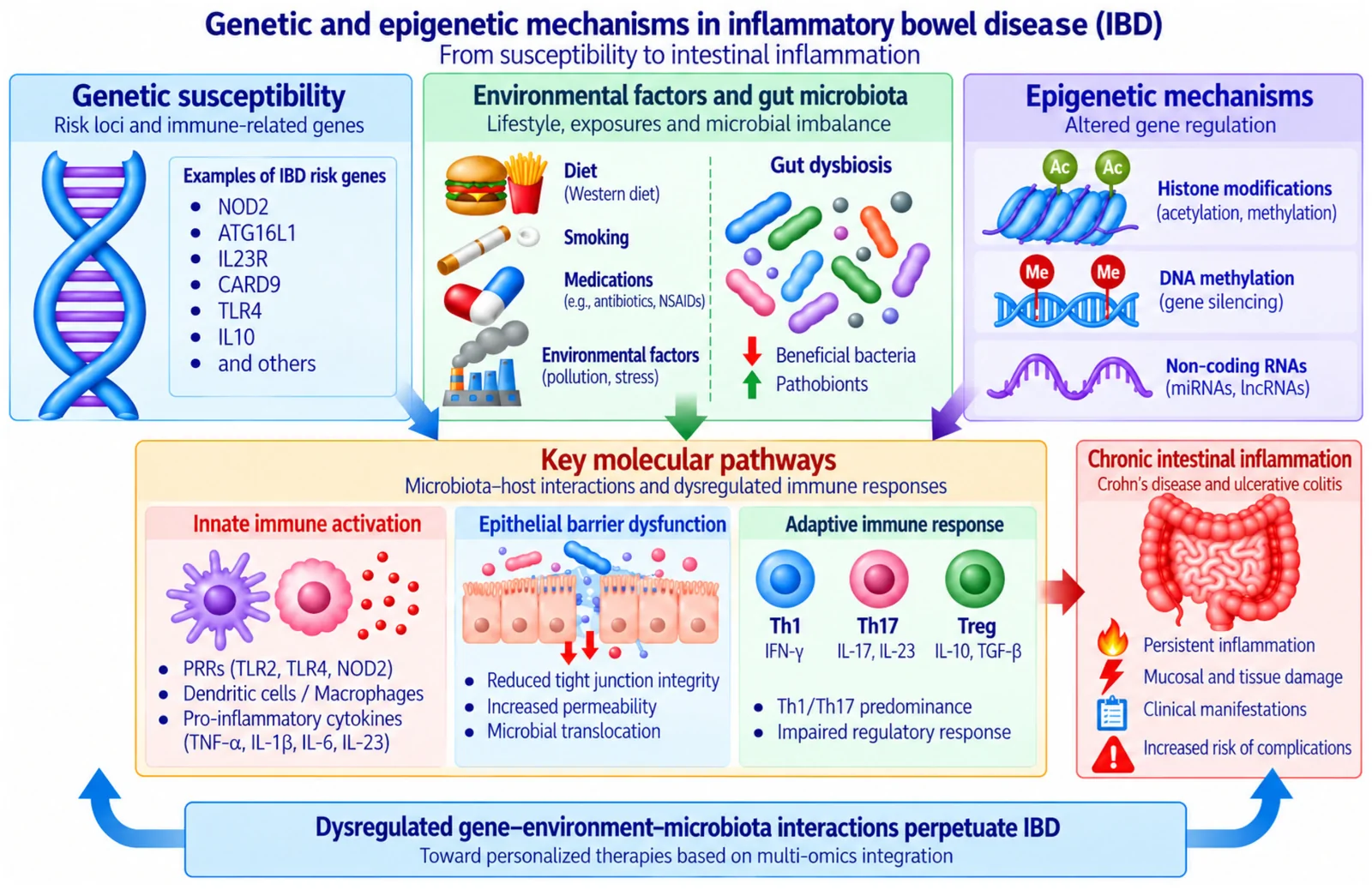
Genetic and epigenetic mechanisms involved in inflammatory bowel disease (IBD). Integrated representation of the interactions among genetic susceptibility, environmental factors, gut microbiota, and epigenetic regulation in IBD. Susceptibility genes, including NOD2, ATG16L1, CARD9, IL23R, and other immune-related genes, converge on pathways involved in microbial sensing, autophagy, epithelial homeostasis, cytokine signaling, and metabolite sensing. Epigenetic mechanisms, including DNA methylation, histone modifications, and non-coding RNAs, further modulate gene expression in response to environmental and microbial signals. These interconnected alterations contribute to epithelial barrier dysfunction, immune dysregulation, and chronic intestinal inflammation, while multi-omics approaches provide opportunities for improved disease stratification and precision medicine. The figure was created using BioRender resources (BioRender.com), accessed on 13 September 2026, with visual refinement for clarity and conceptual and visual assistance from ChatGPT (GPT-6, OpenAI, 2026).

**Figure 6:**
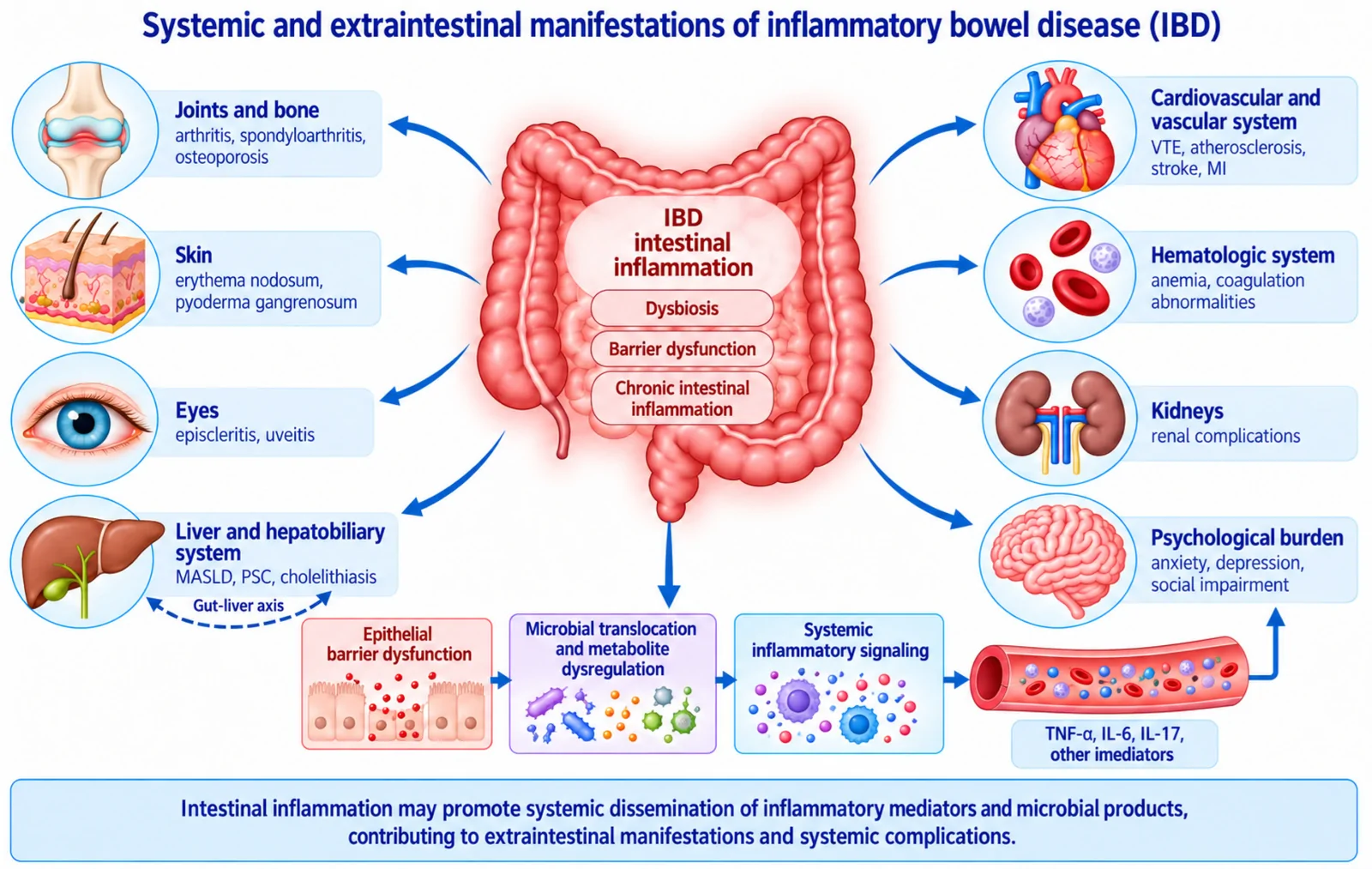
Systemic and extraintestinal manifestations of inflammatory bowel disease (IBD). Overview of the mechanisms linking intestinal inflammation to extraintestinal involvement. Dysbiosis, epithelial barrier dysfunction, microbial translocation, metabolite alterations, and systemic inflammatory signaling may contribute to manifestations involving the musculoskeletal, dermatological, ocular, hepatobiliary, cardiovascular, hematological, renal, and psychological domains. The gut–liver axis represents an important pathway connecting intestinal and hepatobiliary alterations. The figure was created using BioRender resources (BioRender.com), accessed on 13 September 2026, with visual refinement for clarity and conceptual and visual assistance from ChatGPT (GPT-6, OpenAI, 2026).

**Table 2:** Major Alterations in the Gut Microbiota and Their Functional Implications in Inflammatory Bowel Disease.

Microorganism or Taxon	Reported Alteration	Associated Function or Characteristic	Functional Alteration	Potential Relationship with IBD	References
*BACTERIA*					
*Faecalibacterium prausnitzii*	↓	Production of SCFAs, particularly butyrate	Loss	Its depletion may reduce butyrate availability, thereby compromising metabolic support for colonocytes, intestinal barrier integrity, and immunoregulatory mechanisms	[41,56,62]
*Roseburia intestinalis*	↓	Production of SCFAs, particularly butyrate	Loss	Its depletion may reduce butyrate production and impair epithelial and immunoregulatory functions that are important for mucosal homeostasis	[41,56,62]
*Eubacterium rectale*	↓	SCFA production	Loss	Its reduction may contribute to diminished fermentative capacity and lower availability of metabolites associated with intestinal homeostasis	[41]
*Eubacterium hallii*	↓	SCFA production	Loss	Its depletion may reflect the loss of metabolic functions associated with SCFA production	[41]
*Suterella* spp.	↓	Metabolic functions associated with the gut microbiota	Loss	Its depletion is part of the functional alterations described in the IBD-associated microbiota, although its specific contribution to disease progression remains poorly defined	[41]
*Gemmiger formicilis*	↓	SCFA production	Loss	Its depletion may contribute to reduced fermentative capacity and loss of potentially homeostatic metabolic functions	[41]
*Ruminococcus bromii*	↓	Fermentation of complex carbohydrates and indirect contribution to SCFA production	Loss	Its depletion may impair the utilization of dietary substrates and reduce the availability of metabolites derived from microbial fermentation	[41]
*Clostridium* clusters XIVa and IV	↓	SCFA production and amino acid and bile acid metabolism	Loss	Depletion of these groups may impair multiple metabolic pathways involved in maintaining intestinal homeostasis	[41]
*Ruminococcus torques*	↓ in CD	Bile acid metabolism and interaction with the intestinal barrier	Loss/alteration	Its depletion has been observed in pediatric patients with active CD and has been associated with greater disease severity. Experimentally, supplementation improved intestinal barrier markers and modulated bile acid metabolism	[41,69]
*Bifidobacterium* spp.	↓	Amino acid and bile acid metabolism	Loss	Its depletion may contribute to the loss of metabolic functions involved in maintaining intestinal homeostasis	[41]
*Bacteroides* spp.	↓ in some studies; variable according to species and phenotype	Metabolism of complex carbohydrates, amino acids, and bile acids	Alteration	Species-specific changes may affect important metabolic functions within the intestinal microbial community; therefore, a uniform direction of change across the entire genus should be interpreted with caution	[41,57]
*Collinsella aerofaciens*	↓	Iron metabolism	Loss	Its depletion may reflect the loss of microbial metabolic functions, although its direct contribution to IBD progression remains poorly understood	[41]
*Proteobacteria*	↑ predominantly in CD	Association with dysbiosis and the inflammatory environment	Gain/alteration	Expansion of Proteobacteria is frequently associated with intestinal inflammation, although the direction of change may differ between CD and UC	[70]
*Enterobacteriaceae*	↑ in CD; variable in UC	Facultative anaerobes adapted to the inflammatory environment	Gain/alteration	Their expansion may be favored by metabolic and oxidative changes within the inflamed intestine, contributing to the restructuring of the microbial community	[70]
AIEC, adherent-invasive *Escherichia coli*	↑, particularly in CD	Adhesion, invasion, intracellular persistence, and immune activation	Gain	AIEC may induce IL-23 hypersecretion by dendritic cells and promote the conversion of conventional Th17 cells into pathogenic IFN-γ-producing Th17 cells	[71]
*Fusobacterium* spp.	↑	Association with intestinal inflammation	Gain/association	Enrichment of this genus has been associated with IBD and may reflect a microbial environment that favors the persistence of intestinal inflammation	[41]
*Fusobacterium nucleatum*	↑ in CD	Association with inflammatory activity	Gain/association	Greater abundance has been observed in CD and has been positively correlated with fecal calprotectin levels, suggesting an association with increased intestinal inflammatory activity	[72]
*Ruminococcus gnavus*	↑	Production of pro-inflammatory glucorhamnan and utilization of mucin components	Gain	It produces a pro-inflammatory polysaccharide capable of stimulating innate immune cells and increasing TNF production, providing a mechanistic link between bacterial expansion and inflammation	[73]
*Clostridium innocuum*	↑, particularly in association with CD	Association with creeping fat	Gain	It may translocate into mesenteric adipose tissue and has been associated with creeping fat, a characteristic pathological feature of CD	[41]
*Veillonella/Veillonellaceae*	↑	Utilization of metabolites available in the inflammatory environment and participation in microbial networks	Gain/ecological alteration	Its expansion may reflect metabolic conditions favored by inflammation and contribute to ecological restructuring of the intestinal microbiota	[71,74]
*Klebsiella* spp.	Associated with IBD	Pathobiont associated with immune activation	Ecological alteration	It shows increased centrality within microbial networks in patients with IBD, while specific strains have been associated with Th1 responses and intestinal inflammation	[74]
*Enterococcus* spp.	Associated with IBD	Increased resilience in dysbiotic and inflammatory environments	Ecological alteration	Its presence within IBD-associated microbial networks may reflect an enhanced ability to persist under inflammatory conditions without necessarily indicating a uniform increase in abundance	[74]
*FUNGI*					
*Candida albicans*	Predominantly ↑	Pro-inflammatory potential and ability to induce epithelial damage	Gain	Strains with greater damage-inducing capacity may promote epithelial injury and activation of inflammatory responses, although interstudy differences should be considered	[41,75]
*Candida* spp.	Predominantly ↑	Association with inflammation and mycobiome dysbiosis	Gain	Increased abundance of *Candida* is among the most consistent mycobiome alterations reported in both CD and UC	[41,75]
*Saccharomyces cerevisiae*	Variable, frequently ↓ in CD	Immune interactions with the host	Alteration	Changes in abundance are heterogeneous, although depletion has been reported in CD. Cross-reactive T-cell responses to yeasts may also contribute to cytotoxic immune responses in the disease	[41,75,76]
*Debaryomyces hansenii*	↑ in inflamed CD tissue	Impaired mucosal wound healing	Pathological gain	Its enrichment in inflamed or incompletely healed intestinal tissue may impair mucosal repair through a myeloid cell-dependent mechanism involving the type I IFN–CCL5 axis	[41,53]
*Malassezia restricta*	↑ particularly in CD	Association with inflammatory responses	Gain	Its enrichment has been observed particularly in CD, with experimental evidence suggesting that it can exacerbate intestinal inflammation in susceptible hosts	[41,75]
*VIRUSES AND BACTERIOPHAGES*					
Ackermannviridae, particularly *Kuttervirus*	↓	Bacteriophages associated with intestinal bacteria	Loss/ecological alteration	Depletion of Ackermannviridae members has been identified in the intestinal virome of patients with IBD. However, their specific functional contribution to intestinal inflammation remains poorly understood	[77]
*Caudoviricetes*	↑	Major class of tailed bacteriophages within the intestinal ecosystem	Gain/ecological alteration	Their expansion is among the most frequently reported alterations of the IBD virome and accompanies changes in phage–bacterium interactions during dysbiosis	[78,79]
*Siphoviridae*, *Myoviridae*, and *Podoviridae*	↑ in studies using the historical classification	Tailed bacteriophages associated with intestinal bacterial communities	Gain/ecological alteration	Enrichment of these groups has been reported in patients with IBD, particularly in CD tissue, indicating restructuring of the intestinal virome	[41,77]
*Microviridae*	Variable across studies	Bacteriophages associated with intestinal bacterial communities	Ecological alteration	Iliev et al. reported an increase, whereas subsequent reviews and cohort studies have frequently described depletion. The direction of change may depend on disease phenotype and activity, as well as on the methodology used.	[41,79]
*Crassvirales/crAss-like phages*	Predominantly ↓	Abundant bacteriophages within the intestinal microbial ecosystem	Loss/ecological alteration	Their depletion has been associated with altered virome structure in IBD and may reflect reduced stability of interactions between bacteriophages and their bacterial hosts	[79]
*Herelleviridae*, including *Okubovirus*	↑ in specific studies	Bacteriophages associated with Gram-positive bacterial hosts	Gain/ecological alteration	Increased abundance of *Okubovirus* has been reported in samples from patients with IBD, indicating altered bacteriophage community composition; however, its specific causal contribution to intestinal inflammation remains unclear	[41,77]
*Picornaviridae*, including *Enterovirus B*	↑ in specific studies	Eukaryotic viruses associated with the intestinal mucosa	Gain/alteration	*Enterovirus B* enrichment has been reported in colonic tissue from patients with CD and UC, suggesting alteration of the eukaryotic component of the intestinal virome; the contribution of individual taxa to disease progression remains under investigation	[41,77]

Note: ↑, increased relative abundance; ↓, decreased relative abundance.

**Table 3:** Montreal Classification of Ulcerative Colitis According to Disease Extent and Clinical Severity (adapted from Rubin et al. [116]).

Domain	Code	Classification	Description
Extent	E1	Proctitis	Limited to the rectum
	E2	Left-sided colitis	Limited to the colon distal to the splenic flexure
	E3	Extensive colitis	Extends proximal to the splenic flexure
Severity	S0	Remission	Clinical remission without symptoms
	S1	Mild	≤4 bowel movements/day, with or without blood, no systemic signs
	S2	Moderate	≥4 bowel movements/day, with minimal systemic signs of inflammation
	S3	Severe	≥6 bowel movements/day, marked hematochezia, tachycardia ≥ 90 bpm, fever ≥37.5 °C, hemoglobin < 10.5 g/dL, ESR ≥ 30 mm/h

**Table 6:** Mayo Scale for Assessing Ulcerative Colitis Activity (adapted from Pabla and Schwartz [120]).

Component	0 Points	1 Point	2 Points	3 Points
**Stool frequency**	Normal number of bowel movements	1–2 more bowel movements than usual	3–4 more bowel movements than usual	≥5 more bowel movements than usual
**Rectal bleeding**	Absent	Streaks of blood in <50% of bowel movements	Blood evident in most bowel movements	Bleeding in all bowel movements
**Endoscopic Findings**	Mucosa normal, vascular pattern preserved	Mild erythema, mild friability	Marked erythema, absence of vascular pattern, evident friability, erosions	Spontaneous bleeding and diffuse ulceration
**Physician’s global assessment**	Inactive disease	Mild disease	Moderate disease	Severe disease

**Table 7:** Clinical response, loss-of-response, and treatment persistence profiles of major pharmacological therapies for inflammatory bowel disease (IBD).

Therapeutic Class/Representative Agents	Main Use	Clinical Response/Remission Versus Placebo or Active Comparator	Loss-of-Response Profile	Treatment Persistence/Maintenance	References
**Aminosalicylates (5-ASA): mesalazine, sulfasalazine, olsalazine, balsalazide**	Primarily mild-to-moderate UC; limited therapeutic role in CD	During induction, approximately 29% of patients receiving 5-ASA achieved remission versus 17% with placebo. During maintenance, relapse occurred in 37% with 5-ASA versus 55% with placebo over 6–12 months	Primary inadequate response may occur, particularly in more extensive or active disease; dose optimization may be considered in selected patients	Suitable for long-term maintenance in responsive UC; adherence and tolerability substantially influence treatment continuation	[143,144]
**Corticosteroids: prednisone, methylprednisolone, hydrocortisone; budesonide**	Induction of remission in active UC/CD; not recommended for long-term maintenance	In CD, budesonide 9 mg/day was superior to placebo for induction of remission (RR 1.93; 95% CI 1.37–2.73), although less effective than conventional systemic corticosteroids (RR 0.85; 95% CI 0.75–0.97)	Steroid dependence or recurrence during/after tapering indicates insufficient sustained disease control and the need for a steroid-sparing strategy	Budesonide 6 mg/day was not superior to placebo for maintenance (RR 1.13; 95% CI 0.94–1.35); prolonged systemic corticosteroid exposure should be avoided	[145,146]
**Thiopurines: azathioprine and 6-mercaptopurine**	Mainly for maintenance of remission and as steroid-sparing therapy in selected patients with UC/CD	Contemporary reviews support their role in maintenance rather than rapid induction. Placebo-controlled efficacy has been demonstrated in earlier trials summarized in recent reviews, but no recent pivotal placebo-controlled trial provides a directly comparable contemporary response rate	Intolerance, myelotoxicity, hepatotoxicity, pancreatitis, or inadequate disease control may require dose adjustment, discontinuation, or transition to advanced therapy	Long-term maintenance is possible in selected responsive patients, although persistence may be limited by adverse events, monitoring requirements, and availability of targeted therapies	[147]
**Parenteral methotrexate**	Selected patients with moderate-to-severe CD	Parenteral methotrexate is recommended for induction and maintenance therapy in CD; however, direct comparisons with advanced therapies are limited by the absence of modern placebo-controlled pivotal trials reporting comparable efficacy outcomes.	Primary failure, intolerance, or toxicity may limit treatment continuation	Can be used for maintenance in responsive CD; evidence is stronger for parenteral administration than for low-dose oral therapy	[9]
**Anti-TNF: infliximab, adalimumab, golimumab**	Moderate-to-severe UC/CD; induction and maintenance	Infliximab SC, LIBERTY: among patients who responded to IV induction, clinical remission at week 54 occurred in 62.3% with infliximab versus 32.1% with placebo in CD, and 43.2% versus 20.8% with placebo in UC	Secondary loss of response may be associated with low drug exposure, increased clearance, immunogenicity, or anti-drug antibodies, and may require dose intensification or treatment switching	Real-world studies indicate lower persistence of TNF inhibitors than vedolizumab in UC and ustekinumab in CD in selected comparisons	[148,149,150]
**Anti-integrin: vedolizumab**	Moderate-to-severe UC/CD; gut-selective biologic therapy	Active comparator, no placebo: in VARSITY, clinical remission at week 52 was 31.3% with vedolizumab versus 22.5% with adalimumab; endoscopic improvement was 39.7% versus 27.7%, respectively	Pooled incidence of loss of response was 39.8/100 person-years in UC and 47.9/100 person-years in CD; dose intensification restored response in approximately 53.8% of patients with secondary loss of response	Real-world meta-analysis showed 73.8% 1-year persistence in UC, higher than that observed with TNF inhibitors	[150,151,152]
**Anti-IL-12/23: ustekinumab**	Moderate-to-severe UC/CD	In UNIFI, clinical remission at week 8 was 15.5–15.6% with ustekinumab versus 5.3% with placebo. Among induction responders, remission at week 44 was 38.4–43.8% versus 24.0% with placebo withdrawal	Secondary loss of response may occur during maintenance and may require interval shortening or another optimization strategy	Real-world meta-analysis demonstrated 77.5% 1-year persistence in CD, higher than that of TNF inhibitors	[150,151]
**Selective anti-IL-23: risankizumab**	Moderate-to-severe CD	ADVANCE: CDAI remission at week 12 was 45% with risankizumab versus 25% with placebo. MOTIVATE: 42% versus 20% with placebo. In FORTIFY, week-52 remission with 360 mg was approximately 52% versus 41% after withdrawal to placebo	Incomplete or secondary loss of response may occur; treatment optimization may be considered in selected patients	Sustained maintenance efficacy has been demonstrated; comparative long-term real-world persistence data remain less mature than for older biologics	[153,154]
**Selective anti-IL-23: mirikizumab**	Moderate-to-severe UC	In LUCENT, clinical remission at week 12 was 24.2% with mirikizumab versus 13.3% with placebo. Among induction responders, remission at week 40 was 49.9% versus 25.1% with placebo withdrawal	Primary nonresponse or secondary loss of response may occur; extended induction and optimization strategies continue to be evaluated	Maintenance efficacy is established, although comparative real-world persistence data remain limited	[155]
**Selective anti-IL-23: guselkumab**	Moderate-to-severe UC/CD	In QUASAR UC, remission at week 12 was approximately 23% with guselkumab versus 8% with placebo. During maintenance, remission was approximately 45–50% versus 19% with placebo withdrawal. Phase 3 GALAXI studies also demonstrated substantial efficacy in CD	Long-term patterns of secondary loss of response and optimal rescue strategies remain under investigation	Maintenance efficacy has been demonstrated; however, mature real-world persistence data remain limited because of its relatively recent introduction into clinical practice	[156,157]
**JAK inhibitor: tofacitinib**	Moderate-to-severe UC	OCTAVE Induction 1: remission at week 8 was 18.5% with tofacitinib versus 8.2% with placebo; OCTAVE Induction 2: 16.6% versus 3.6% with placebo. At week 52, remission was 34.3% with 5 mg BID and 40.6% with 10 mg BID versus 11.1% with placebo withdrawal	Secondary loss of response may occur; anti-drug antibody formation is not applicable to this small-molecule therapy	Sustained maintenance is possible; continuation depends on efficacy, dose requirements, and individualized safety assessment	[158]
**JAK inhibitor: upadacitinib**	Moderate-to-severe UC/CD; quantitative data shown here refer to UC	U-ACHIEVE: remission at week 8 was 26.1% with upadacitinib versus 4.8% with placebo. U-ACCOMPLISH: 33.5% versus 4.1% with placebo. At week 52, remission was 42.3% with 15 mg and 51.7% with 30 mg versus 12.1% with placebo withdrawal	Secondary loss of response may occur; immunogenicity is not applicable. Dose adjustment may be considered according to clinical response and safety	Strong maintenance efficacy has been demonstrated; real-world evidence on treatment persistence is increasing	[159]
**S1P receptor modulator: ozanimod**	Moderate-to-severe UC	In True North, clinical remission at week 10 was 18.4% with ozanimod versus 6.0% with placebo. Among maintenance responders, remission at week 52 was 37.0% versus 18.5% with placebo withdrawal	Loss of response may occur during maintenance; immunogenicity is not applicable	Maintenance efficacy has been demonstrated, although comparative real-world persistence data remain less established than for older biologics	[160]
**S1P receptor modulator: etrasimod**	Moderate-to-severe UC	ELEVATE UC 52, treat-through design: remission was 27% with etrasimod versus 7% with placebo at week 12 and 32% versus 7% with placebo at week 52. ELEVATE UC 12: 25% versus 15% with placebo at week 12	Primary incomplete response or secondary loss of disease control may occur; immunogenicity is not applicable	Continuous treatment demonstrated maintenance of efficacy through week 52; real-world persistence data are still emerging	[161]

Note: Clinical response and remission estimates should not be directly compared across therapies because pivotal trials differ substantially in patient populations, previous exposure to biologic or targeted therapies, endpoint definitions, dosing regimens, and assessment time points. In randomized-withdrawal maintenance trials, indicated in the table as “placebo withdrawal,” patients initially responded to active induction therapy and were subsequently randomized either to continue active treatment or to receive placebo; therefore, the placebo group does not represent untreated patients from study initiation. In VARSITY, the comparator was adalimumab rather than placebo. ELEVATE UC 52 used a continuous treat-through design.

## Data Availability

No new data were created or analyzed in this study. Data sharing is not applicable to this article.

## Abbreviations

The following abbreviations are used in this manuscript:

5-ASA	5-Aminosalicylic Acid
AhR	Aryl Hydrocarbon Receptor
AIEC	Adherent-Invasive *Escherichia coli*
anti-TNF	Anti-Tumor Necrosis Factor
ATP	Adenosine Triphosphate
ATG16L1	Autophagy-Related 16 Like 1
BID	Twice Daily
CARD9	Caspase Recruitment Domain Family Member 9
CCL5	C-C Motif Chemokine Ligand 5
CCL20	C-C Motif Chemokine Ligand 20
CCR5	C-C Chemokine Receptor Type 5
CCR6	C-C Chemokine Receptor Type 6
CD	Crohn’s Disease
CDAI	Crohn’s Disease Activity Index
CI	Confidence Interval
CLEC5A	C-Type Lectin Domain Containing 5A
CLEC7A	C-Type Lectin Domain Family 7 Member A
CRP	C-Reactive Protein
CTE	Computed Tomography Enterography
DCA	Deoxycholic Acid
DeCS	Descritores em Ciências da Saúde
DNA	Deoxyribonucleic Acid
DUOX2	Dual Oxidase 2
DUOXA2	Dual Oxidase Maturation Factor 2
EIMs	Extraintestinal Manifestations
ESR	Erythrocyte Sedimentation Rate
GIT	Gastrointestinal Tract
GPCRs	G Protein-Coupled Receptors
H_2_O_2_	Hydrogen Peroxide
Hct	Hematocrit
HDACs	Histone Deacetylases
HIF-1α	Hypoxia-Inducible Factor 1-Alpha
HK2	Hexokinase 2
IBD	Inflammatory Bowel Disease
IBD-U	Inflammatory Bowel Disease Unclassified
IFN	Interferon
IFN-γ	Interferon Gamma
IL-1β	Interleukin-1 Beta
IL-6	Interleukin-6
IL-10	Interleukin-10
IL-12	Interleukin-12
IL-17	Interleukin-17
IL17A	Interleukin-17A
IL-22	Interleukin-22
IL-23	Interleukin-23
IL23R	Interleukin-23 Receptor
IRGM	Immunity-Related GTPase M
IV	Intravenous
JAK	Janus Kinase
LCA	Lithocholic Acid
MAPK	Mitogen-Activated Protein Kinase
MASLD	Metabolic Dysfunction-Associated Steatotic Liver Disease
MeSH	Medical Subject Headings
miRNAs	MicroRNAs
mRNA	Messenger RNA
MRE	Magnetic Resonance Enterography
mtROS	Mitochondrial Reactive Oxygen Species
mTOR	Mechanistic Target of Rapamycin
NOD2	Nucleotide-Binding Oligomerization Domain Containing 2
OXPHOS	Oxidative Phosphorylation
PCDAI	Pediatric Crohn’s Disease Activity Index
PGC-1α	Peroxisome Proliferator-Activated Receptor Gamma Coactivator 1-Alpha
PTPN2	Protein Tyrosine Phosphatase Non-Receptor Type 2
PUCAI	Pediatric Ulcerative Colitis Activity Index
RASSF1A	Ras Association Domain Family Member 1, Isoform A
RNA	Ribonucleic Acid
ROS	Reactive Oxygen Species
RR	Risk Ratio
S100A12	S100 Calcium Binding Protein A12
S1P	Sphingosine-1-Phosphate
SC	Subcutaneous
SCFAs	Short-Chain Fatty Acids
SLC22A4	Solute Carrier Family 22 Member 4
SLC22A5	Solute Carrier Family 22 Member 5
TFEB	Transcription Factor EB
Th1	T Helper 1
Th2	T Helper 2
Th17	T Helper 17
TLR4	Toll-Like Receptor 4
TNF	Tumor Necrosis Factor
TNF-α	Tumor Necrosis Factor Alpha
Treg	Regulatory T Cell
UC	Ulcerative Colitis
WT	Wild Type
